# Chemosensitivity of Patient-Derived Cancer Stem Cells Identifies Colorectal Cancer Patients with Potential Benefit from FGFR Inhibitor Therapy

**DOI:** 10.3390/cancers12082010

**Published:** 2020-07-22

**Authors:** Takehito Yamamoto, Hiroyuki Miyoshi, Fumihiko Kakizaki, Hisatsugu Maekawa, Tadayoshi Yamaura, Tomonori Morimoto, Toshiro Katayama, Kenji Kawada, Yoshiharu Sakai, M. Mark Taketo

**Affiliations:** 1 Division of Experimental Therapeutics, Graduate School of Medicine, Kyoto University, Kyoto 606-8501, Japan; takehito_y@kuhp.kyoto-u.ac.jp (T.Y.); hmiyoshi@mfour.med.kyoto-u.ac.jp (H.M.); f-kakizaki@mfour.med.kyoto-u.ac.jp (F.K.); hisatsug@kuhp.kyoto-u.ac.jp (H.M.); tyamaura@kuhp.kyoto-u.ac.jp (T.Y.); tmori@kuhp.kyoto-u.ac.jp (T.M.); 2 Institute for Advancement of Clinical and Translational Science (iACT), Kyoto University Hospital, Sakyo-ku, Kyoto 606-8507, Japan; 3 Departments of Surgery, Graduate School of Medicine, Kyoto University, Sakyo-ku, Kyoto 606-8507, Japan; kkawada@kuhp.kyoto-u.ac.jp (K.K.); ysakai@kuhp.kyoto-u.ac.jp (Y.S.); 4 Office of Society-Academia Collaboration for Innovation, Kyoto University, Sakyo-ku, Kyoto 606-8501, Japan; 5 Kitano Hospital, The Tazuke Kofukai Medical Research Institute, Kita-ku, Osaka 530-8480, Japan; prof.katayama@gmail.com

**Keywords:** fibroblast growth factor receptor (FGFR) inhibitor, chemosensitivity, cancer stem cell, spheroid, organoid, patient-derived xenograft (PDX)

## Abstract

Some colorectal cancer patients harboring *FGFR* (fibroblast growth factor receptor) genetic alterations, such as copy number gain, mutation, and/or mRNA overexpression, were selected for enrollment in several recent clinical trials of FGFR inhibitor, because these genetic alterations were preclinically reported to be associated with FGFR inhibitor sensitivity as well as poor prognosis, invasiveness, and/or metastatic potential. However, few enrolled patients were responsive to FGFR inhibitors. Thus, practical strategies are eagerly awaited that can stratify patients for the subset that potentially responds to FGFR inhibitor chemotherapy. In the present study, we evaluated the sensitivity to FGFR inhibitor erdafitinib on 25 patient-derived tumor-initiating cell (TIC) spheroid lines carrying wild-type *RAS* and *RAF* genes, both in vitro and in vivo. Then, we assessed possible correlations between the sensitivity and the genetic/genomic data of the spheroid lines tested. Upon their exposure to erdafitinib, seven lines (7/25, 28%) responded significantly. Normal colonic epithelial stem cells were unaffected by the inhibitors. Moreover, the combination of erdafitinib with EGFR inhibitor erlotinib showed stronger growth inhibition than either drug alone, as efficacy was observed in 21 lines (84%) including 14 (56%) that were insensitive to erdafitinib alone. The in vitro erdafitinib response was accurately reflected on mouse xenografts of TIC spheroid lines. However, we found little correlation between their genetic/genomic alterations of TIC spheroids and the sensitivity to the FGFR inhibitor. Accordingly, we propose that direct testing of the patient-derived spheroids in vitro is one of the most reliable personalized methods in FGFR-inhibitor therapy of colorectal cancer patients.

## 1. Introduction

The fibroblast growth factor (FGF) ligand family, composed of 22 protein ligand members, is one of the direct promoter proteins of endothelial cell proliferation, affecting many types of normal cells as well as cancer cells [1,2]. As FGF signaling plays a crucial role in tumor cell proliferation, migration, and survival [3,4], FGFs and their receptors (fibroblast growth factor receptors; FGFRs) are considered as druggable therapeutic targets. The FGFR family contains four receptor tyrosine kinase paralogs (FGFR1–4) to which FGF ligands can bind [2].

Several sets of preclinical data showed significant growth inhibition by FGFR small-molecule inhibitors on cancer cell lines or xenografts that had *FGFR* gene amplifications [5,6,7,8]. Accordingly, multiple clinical Phase I trials of FGFR inhibitors were performed on patients with cancers carrying such alterations [9,10,11,12,13,14,15,16,17]. Except for urothelial cancers with *FGFR3* mRNA overexpression [17], FGFR inhibitors did not always improve patient survival compared with conventional treatments [9,10]. This rather poor response to FGFR inhibitors was hypothesized by intratumor heterogeneity of *FGFR* aberrations and/or by the discrepancy between gene copy number gain and expression levels of *FGFR* mRNA or protein [9,10]. Even in the urothelial cancer trial [17]; however, about one-quarter of enrolled patients with *FGFR* mRNA overexpression did not respond to the drug. On the other hand, a small subset of patients in these clinical trials showed significantly better (complete or partial) response to the FGFR inhibitors than others, regardless of *FGFR* gene copy numbers or mRNA expression levels.

There have been no clinical trials or preclinical studies exclusively focusing on FGFR inhibitors for colorectal cancer patients likely because the frequency of *FGFR* aberration (amplification) is only ~4% in this type of cancer [3]. However, several basket studies for different types of solid tumors included small numbers of colorectal cancer patients. For example, a Phase I clinical trial for FGFR1–3 inhibitor AZD4547 for 30 solid cancer patients with *FGFR* mutations, amplifications, or fusions included three colorectal cancer patients, but no data on their sensitivity to AZD4547 were available [11]. Another Phase I study for FGFR1–3 inhibitor infigratinib for 132 solid cancer patients with *FGFR* gene alterations, enrolled five colorectal patients, and two of them were reported “resistant” to infigratinib [13]. Moreover, a Phase I–II trial for pan-FGFR inhibitor erdafitinib for 19 solid cancer patients had three colorectal cancer patients, but no data on individual patient sensitivity were disclosed [12]. No detailed data in these studies were shown regarding *FGFR* gene aberrations in each colorectal cancer tissue. Meanwhile, a Phase I study for FGFR1–3 inhibitor rogaratinib investigated 866 solid cancer patients, including 46 colorectal cancer patients, and showed that the tumor of only one patient had *FGFR* mRNA overexpression. Accordingly, this particular patient was enrolled in the dosing study, but no data on the sensitivity were given [17]. Collectively, referring to previous reports, there were few data on the prevalence of *FGFR* gene aberrations or their relationship with FGFR inhibitor sensitivity among colorectal cancer patients. This is because a Phase I trial is designed to study the safety and dose ranges, and the efficacy information is not always obtained.

Recently, culturing patient-derived tumor-initiating cell (TIC) spheroids (or, organoids) and their xenografts (patient-derived spheroid xenografts; PDSXs) have attracted attention for possible therapeutic development acceleration by drug screening [18,19]. We previously reported the method of establishing patient-derived spheroids with a success rate of over 90% within approximately 1–3 weeks [20]. We also demonstrated that the drug sensitivity in PDSXs of primary tumors accurately reflects the clinical chemosensitivity of colorectal cancer patients and indicated that PDSXs are superior to the conventional PDXs because of smaller variances in tumor volume and higher reproducibility and, therefore, more suitable for drug efficacy evaluation [21].

Although there were several papers that reported the efficacy of FGFR inhibitors on colorectal cancer cell lines or small numbers of PDXs [7,22,23], few of them focused on patient-derived TIC spheroids. Here, we addressed this topic by designing and validating a protocol using patient-derived spheroids to identify colorectal cancer patients who could benefit from FGFR inhibitor therapies. We found that there was a discrepancy between the sensitivity of colorectal cancer spheroids against FGFR inhibitors and their genetic/genomic aberration status, and that assessment of chemosensitivity directly on spheroids is a better correlated strategy that helps select colorectal cancer patient subpopulations that can benefit from FGFR inhibitors.

## 2. Results

### 2.1. The in vitro Evaluation of the FGFR Inhibitors for Colorectal Cancer Pateints by Using Patient-Derived TIC Spheroids

We investigated a total of 25 colorectal cancer patient-derived TIC spheroid lines for sensitivity to pan-FGFR inhibitor erdafitinib as well as EGFR inhibitor, erlotinib. All 25 lines were *RAS/RAF*-wild type. In *RAS/RAF*-activating mutant colorectal cancer, RAS or RAF constitutively stimulates the MAPK pathway downstream. Accordingly, the upstream blockade at the level of FGFR or EGFR is essentially ineffective [24]. In fact, anti-EGFR antibodies, cetuximab and panitumumab, are prescribed exclusively for wild-type *RAS/RAF* colorectal cancer patients, not for *RAS/RAF* mutants. Accordingly, we investigated *RAS/RAF* wild-type cancer patients exclusively in the present study.

Table 1 summarizes the clinicopathological characteristics, mutational statuses, and responses to erdafitinib, erlotinib, and the combination of both, for the patients enrolled in this study. Notably, seven of the 25 spheroid lines were sensitive to erdafitinib single treatment (28%; 95% C.I. 11–46%), whereas five of 25 (20%; 95% C.I. 5–35%) were sensitive to erlotinib singly (Table 1). The former result suggests that approximately one-third of the patients with *RAS/RAF* wild-type colorectal cancer may benefit from FGFR inhibitor monotherapy. Importantly, 21 of 25 *RAS/RAF* wild-type spheroid lines (84%; 95% C.I. 70–98%) responded to the combination treatment, suggesting the possibility that a majority of *RAS/RAF* wild-type colorectal cancer patients were sensitive to the combination therapy.

We describe experimental details in the following sections.

### 2.2. FGFR Inhibitors Suppress in vitro Growth of Colorectal Cancer TIC Spheroids Derived from a Subset of Patients

**Figure 1:**
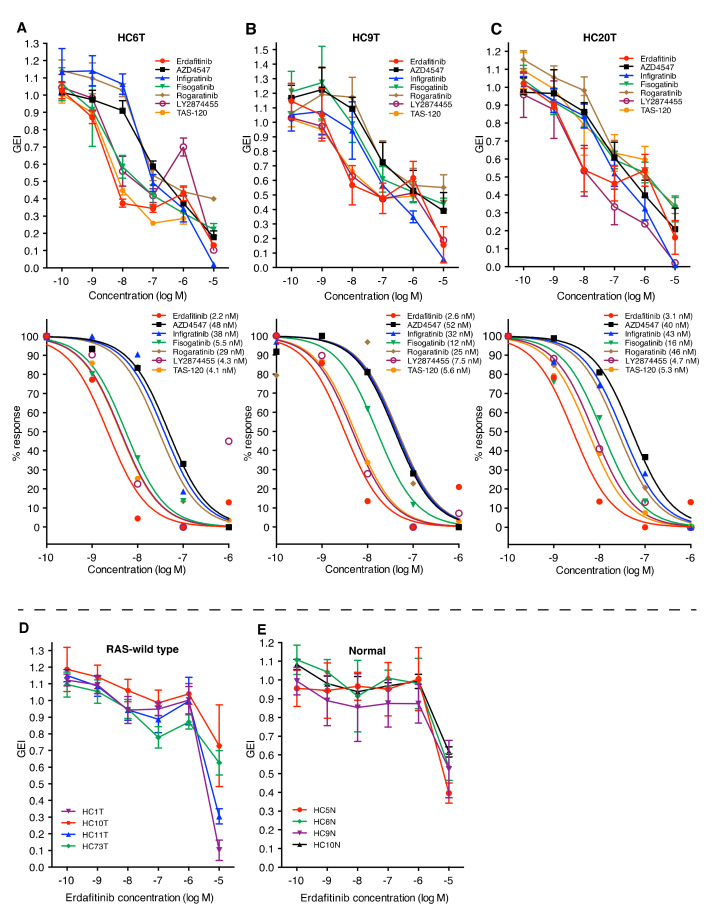
Effects of FGFR inhibitors on the in vitro growth of colorectal cancer TIC (tumor-initiating cell) spheroids. (**A**–**C**), Dose-dependent effects (top; raw data) and computer-fitted dose–response curves (bottom) of seven FGFR inhibitors (erdafitinib, AZD4547, infigratinib, fisogatinib, rogaratinib, LY2874455, and TAS-120) on the growth of three colorectal cancer TIC spheroid lines: HC6T (**A**), HC9T (**B**), and HC20T (**C**). Luciferase-expressing spheroid cells were cultured for three days with indicated concentrations of drugs, and their relative growth rates (growth effect indices; GEI) were calculated from the photon counts. The IC_50_ value is also shown in parentheses for each drug. The data at the concentration of 10^-5^ M (10 μM) were excluded from the computation of the best-fit dose–response curves because of their non-specific nature of the toxicity (see also **D**,**E**). Some dose–response curves overlapped in HC6T (LY2874455 and TAS-120) and HC9T (AZD4547, infigratinib and rogaratinib). (**D**) Effects of erdafitinib on the growth of another set of *RAS/RAF* wild-type colorectal cancer spheroid lines, HC1T, HC10T, HC11T, and HC73T. (**E**), Effects of erdafitinib on the growth of four normal colonic epithelial stem cell spheroid lines (HC5N, HC6N, HC9N, HC10N). All sets of data show the mean values ± SD of three independent experiments each of which was performed in four replicates.

We recently reported that colorectal cancer TIC spheroids from some patients depended on basic fibroblast growth factor (bFGF; FGF2) for their proliferation in vitro [20], which led us to hypothesize that FGFR inhibitors might be efficacious for a subset of colorectal cancer patients. To test the hypothesis, we first treated three such spheroid lines (HC6T, HC9T, and HC20T; Table 1) with seven small-molecule FGFR inhibitors—erdafitinib (JNJ42756493), AZD4547, infigratinib (BGJ398), fisogatinib (BLU554), rogaratinib (BAY1163877), LY2874455, and TAS-120—and determined their growth effect indices (GEI; the growth rates of treated spheroids relative to those with solvent control, see Section 4) [20]. These spheroid lines contained no activating mutations in *KRAS*, *NRAS*, and *BRAF* genes (Table S1), and growth of the former two lines was heavily dependent on bFGF [20]. As shown in Figure 1A–C, all seven FGFR inhibitors suppressed the growth of these spheroid lines in a dose-dependent manner. Among them, erdafitinib (a pan-inhibitor of FGFR1–4) was the most potent against the three spheroid lines (IC_50_, 2.2–3.1 nM).

The result that FGFR4-selective inhibitor fisogatinib inhibited the growth of these spheroid lines indicated that, at least, FGFR4 played a key role among the four receptor paralogs. At the same time, the pan-FGFR inhibitor erdafitinib suppressed spheroid proliferation 2–5 times more strongly than fisogatinib, suggesting that the other receptor paralogs were also involved in the growth of these TIC spheroids (Figure 1A–C).

We then tested other spheroid lines that did not depend on bFGF for in vitro growth. Notably, erdafitinib showed little growth inhibition on four such spheroid lines (HC1T, HC10T, HC11T, and HC73T) at clinically relevant doses (0.01–0.1 μM) despite that they carried wild-type *RAS* and *RAF* genes (Figure 1D). These results suggested that their proliferation was supported by other growth factor receptors (e.g., EGFR; see below). Importantly, these FGFR inhibitors hardly suppressed the growth of normal colorectal epithelial stem cell spheroids, derived from multiple patients at concentrations below 1 μM (Figure 1E), predicting few mechanism-based adverse effects on the normal colonic mucosa.

### 2.3. Synergistic in vitro Growth Suppression by Combination Treatment with FGFR/EGFR Inhibitors

As the efficacy of FGFR inhibitors was insufficient to completely halt the proliferation of TIC spheroids (50–70% decrease in growth, Figure 1A–C) and because the downstream signaling pathway of FGFR is shared by that of EGFR [1], we speculated that simultaneous inhibition of FGFR and EGFR could act in a synergistic manner. Among the EGFR inhibitors, monoclonal antibodies cetuximab and panitumumab have already been approved for treatment of *RAS/RAF* wild-type colorectal cancer [25,26]. However, cetuximab failed to block the in vitro growth of the three spheroid lines tested against the seven FGFR inhibitors, likely because of its poor permeability to the culture medium that contained Matrigel (Figure S1A, right). Accordingly, we instead employed the EGFR small-molecule inhibitor erlotinib of which efficacy for colorectal cancer was proved but not approved for clinical use due to the adverse events severer than cetuximab [27,28]. As anticipated, erlotinib caused dose-dependent growth inhibition of spheroid lines, HC6T, HC9T, and HC20T. (Figure S1A, left).

**Figure 2:**
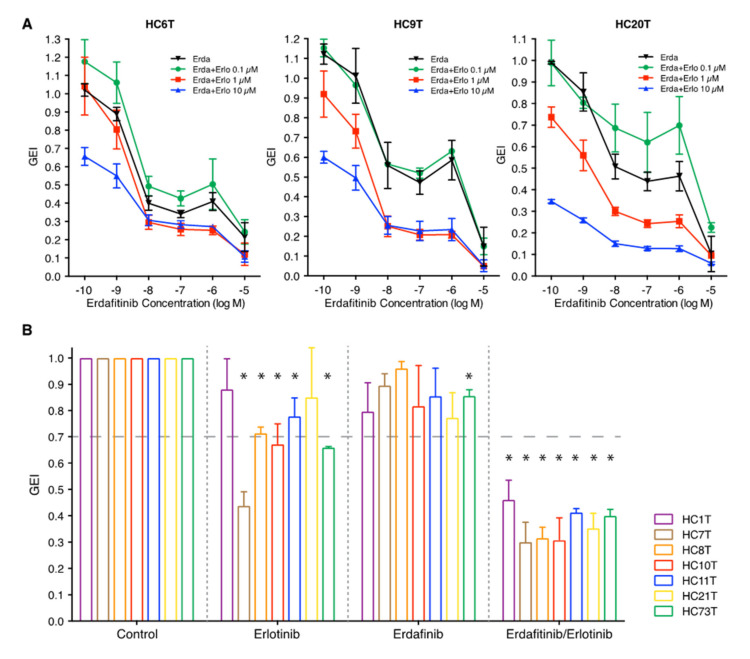
Synergistic effects of FGFR and EGFR inhibition on the growth of colorectal cancer TIC (tumor-initiating cell) spheroids. (**A**) Dose-dependent inhibitory effects of erdafitinib (FGFR inhibitor) in the absence or presence of erlotinib (EGFR inhibitor) at three different concentrations (0.1, 1, and 10 μM) on the growth of three colorectal cancer spheroid lines (HC6T, left; HC9T, center; and HC20T, right). (**B**) The inhibitory effects of erdafitinib alone (0.1 μM), erlotinib alone (1 μM), or combination of both on seven spheroid lines that were free of *RAS/RAF*-activating mutations (HC1T, HC7T, HC8T, HC10T, HC11T, HC21T, and HC73T). The cut-off GEI between “responsive” and “non-responsive” was assigned at 0.7 (70%). All sets of data show the mean values ± SD of three independent experiments each of which was performed in four replicates. The statistical significance of the data difference is shown; *, *p* < 0.05 in one-way ANOVA followed by Tukey’s post-test.

We then tested the combined effects of erdafitinib and erlotinib on the same three spheroid lines (Figure 2A). Growth suppression was enhanced in the presence of both inhibitors compared with either inhibitor alone, at clinically relevant doses of 0.01–0.1 μM erdafitinib and 1 μM erlotinib (HC6T, *p* < 0.05; HC9T and HC20T, *p* < 0.01; Figure 2A).

We consequently asked whether other *RAS/RAF* wild-type spheroid lines that were insensitive to erdafitinib alone showed sensitivity to the combination of erdafitinib/erlotinib (HC1T, HC7T, HC8T, HC10T, HC11T, HC21T, and HC73T; Figure 2B). Interestingly, all seven lines responded to the combination treatment (erdafitinib at 0.1 μM and erlotinib at 1 μM), suppressing the growth to less than 50% of GEI (*p* < 0.05, Figure 2B). In addition to these lines and the three erdafitinib-sensitive lines (Figure 1A–C), we tested the remaining 15 *RAS/RAF* wild-type spheroid lines with the combination of inhibitors as well as with each inhibitor separately (Figure S1B). Among them, 11 of 15 responded to the combination (GEI < 70%) including four lines that were insensitive to erlotinib alone and two lines not responding to erdafitinib alone. On the other hand, the growth of normal colorectal epithelial stem cell spheroids (HC6N and HC10N) were hardly suppressed by combination of the two inhibitors (Figure S1B).

### 2.4. Immunoblotting Analysis of Colorectal Cancer Spheroid Lines for FGFR, EGFR, and Downstream Effector Proteins

**Figure 3:**
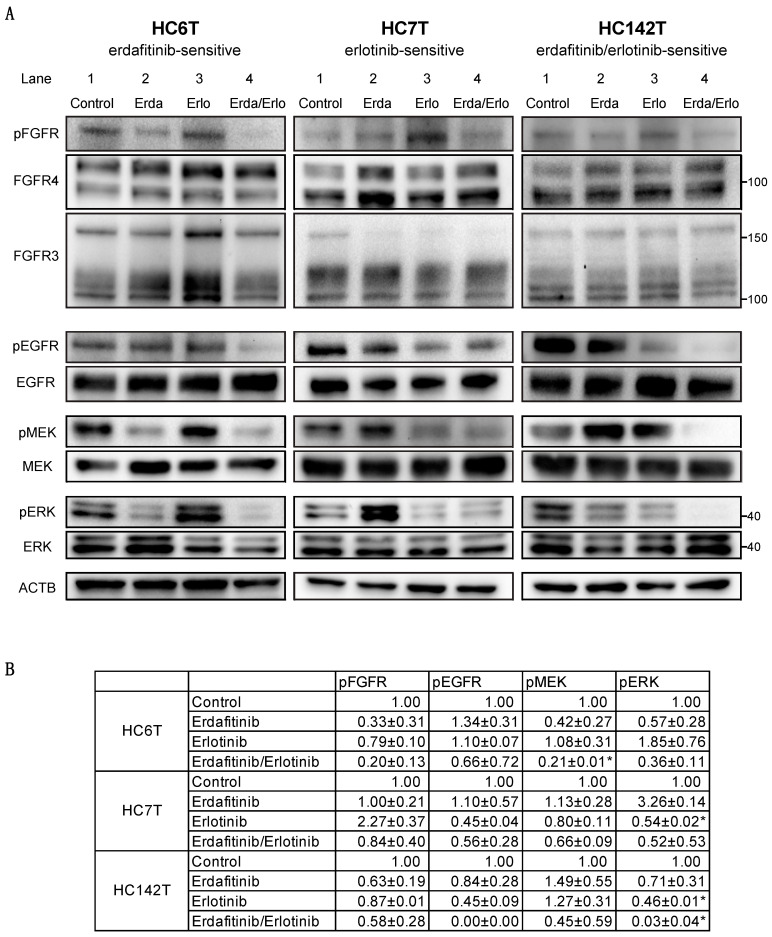
Immunoblotting analysis of colorectal cancer spheroid lines for FGFR, EGFR, and downstream effector proteins. (**A**) Left, HC6T responsive to erdafitinib alone; center, HC7T responsive to erlotinib alone; and right, HC142T responsive to erdafitinib/erlotinib combination, but non-responsive to either of them alone. Control, no-drug control; Erda, erdafitinib; Erlo, erlotinib; and Erda/Erlo, combination of erdafitinib and erlotinib. Antibodies were used to detect pFGFR, for FGFR phosphorylated at Y653/654; FGFR4 and FGFR3, for FGFR4 and FGFR3 proteins, respectively; pEGFR for EGFR phosphorylated at Y1068; EGFR for EGFR protein; pMEK for MEK phosphorylated at S217/221; MEK for MEK protein; pERK for ERK phosphorylated at T202/221; ERK for ERK protein; and ACTB for β-actin protein for calibration of the amount of total protein loaded onto each lane. The positions of some molecular weight markers are shown on the right margin as *M*r ×10^−3^ Da. The uppermost band of ~165 kD in FGFR3 represents the receptor molecule still holding the extracellular domain that tends to be lost in experimental procedures [29,30]. Uncropped images of the Western Blot can be found at Figure S7. (**B**) Quantified data of gel images in immunoblotting analysis of colorectal cancer spheroid lines for FGFR, EGFR, and downstream effector proteins. The values were normalized to those of protein amounts and presented as the proportionally relative to the solvent control. Each value is presented as the mean ± SD, and a significant reduction in phosphorylation is indicated with asterisk; * *p* < 0.05 in paired T-test.

To investigate the molecular mechanism behind the responses to erdafitinib and/or erlotinib on the colorectal cancer TIC spheroids, we determined the levels of expression and phosphorylation for key signaling proteins by Western blotting analysis using three representative spheroid cell lines. In erdafitinib-sensitive/erlotinib-resistant HC6T, tyrosine residues (Y653 and Y654) in FGFR1–4 were phosphorylated (pFGFR) when untreated; phosphorylation was reduced by erdafitinib although the total protein abundance of FGFR 3/4 was unaffected (Figure 3A; upper left, lanes 1 and 2, and Figure 3B; *FGFR 1/2* mRNA was scarcely expressed and therefore not examined; see below). Consistently, the two downstream proteins MEK and ERK were also phosphorylated at S217/221 (pMEK) and T202/Y204 (pERK), respectively, which was inhibited by treatment with erdafitinib (Figure 3A, left, lane 2, and Figure 3B). On the other hand, erlotinib alone did not reduce phosphorylation of FGFR3/4 (pFGFR) or downstream MEK/ERK (pMEK/pERK; Figure 3A, left, lane 3, and Figure 3B). Combination of erdafitinib and erlotinib inhibited such phosphorylation at extents similar to, but slightly stronger than, erdafitinib alone (Figure 3A, left, lane 4, and Figure 3B). These results were consistent with the erdafitinib sensitivity of this spheroid line in vitro (Figure 1 and Table 1).

In erdafitinib-resistant/erlotinib-sensitive HC7T, FGFR phosphorylation at Y653/654 (pFGFR) was too weak to be detected despite substantial levels of FGFR 3 and 4 protein (Figure 3A; center, lane 1). By erlotinib, the level of phosphorylation was reduced in MEK and moderately in ERK1/2 (lane 3). As pMEK and pERK1/2 were at similar levels between erlotinib alone and in combination with erdafitinib, inhibition of phosphorylation by this combination could be caused essentially by erlotinib (Figure 3A; center, lanes 3, 4, and Figure 3B). Notably, the level of pERK1/2 and pMEK in HC7T was increased by erdafitinib alone compared with the no-drug control. This observation may be explained by feedback and compensatory response caused by redundancy in FGFR and EGFR signaling [31]. As anticipated, levels of the MEK or ERK1/2 proteins were not affected substantially by these drugs. Thus, these results are also consistent with those of in vitro chemosensitivity of this spheroid line (Table 1, Figure 2B).

Finally, in HC142T that was sensitive to the erdafitinib/erlotinib combination but insensitive to either of them alone, the phosphorylation level of FGFR was weaker than that in erdafitinib-sensitive HC6T (Figure 3A, right, lane 1). Both erdafitinib alone and combination of the two drugs weakened the pFGFR band (Figure 3A, right, lane 2, 4). The effects of these drugs were more evident when phosphorylation was analyzed for downstream MEK and ERK1/2, being reduced significantly by the combination treatment (Figure 3A, right, lane 4, and Figure 3B). Interestingly, the level of pMEK rather increased by either drug alone, whereas that of pERK1/2 was decreased compared with the level in the control, which was likely because of the feedback and compensatory mechanism as described above [31]. Although the protein level of FGFR4 appeared comparable with that in HC6T, the relative strength of the two bands for FGFR4 were rather reciprocal between the two spheroid lines. Namely, the top band representing the terminally glycosylated form [32] was stronger in HC6T, whereas the bottom one for the core-glycosylated form was dominant in HC7T as well as in HC142T (Figure 3A).

These results taken together suggested that the relative signaling strengths of FGFR and EGFR were correlated with individual TIC response to the small molecule inhibitors.

### 2.5. Erdafitinib and/or Cetuximab Reduce the Size of Patient-Derived Spheroid-Xenograft (PDSX) Tumors at Clinically Relevant Doses

To determine the potential in vivo efficacy of drugs that block FGFR and/or EGFR signaling, we established patient-derived spheroid xenografts (PDSXs) in immunocompromised mice from spheroid lines HC6T and HC20T that responded well to not only FGFR inhibitors singly but also to FGFR/EGFR inhibitor combination treatments (Figure 1A,C, Figure 2A and Figure S2); we further established PDSXs from spheroid line HC1T that was resistant to erdafitinib or erlotinib alone (Table 1, Figure 2B, and Figure S2). This in vivo PDSX model is useful for drug evaluation as we verified by a retrospective clinical study [21]. We divided PDSX mice into four treatment groups; control (solvent only), erdafitinib and cetuximab monotherapies, and erdafitinib/cetuximab combination. For chemotherapy of *RAS/RAF* wild-type colorectal cancer patients, cetuximab and panitumumab are used rather than erlotinib [24]. Therefore, cetuximab was adopted in the in vivo PDSX experiments.

**Figure 4:**
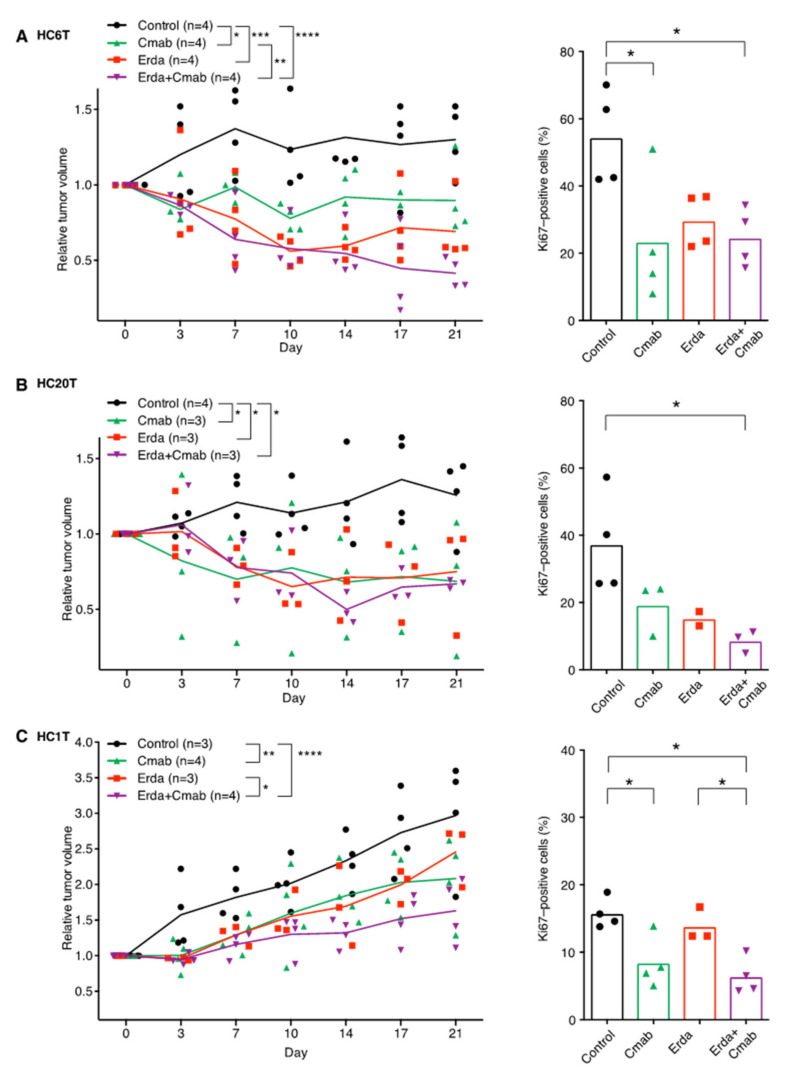
Drug-sensitivity tests with erdafitinib and/or cetuximab against PDSX-tumor growth in mice. (**A–C**), left. Changes in the relative subcutaneous tumor volumes of HC6T (**A**), HC20T, (**B**) and HC1T (**C**) xenografts during drug treatments. (**A–C**), right. The fraction of proliferating cells (%) monitored by Ki67 expression for each treatment group. The mean values are shown as bars with raw data points in symbols of various shape. Among HC20T PDSX mice, one of the three tumors in the erdafitinib monotherapy group disappeared (complete response), and it is not included in the figure (**B**, right). The statistical significance of the data difference is shown; *, *p* < 0.05; **, *p* < 0.01; ***, *p* < 0.001; or ****, *p* < 0.0001; in one-way or two-way ANOVA followed by Tukey’s post-test.

In PDSX mice with HC6T tumors, erdafitinib and cetuximab monotherapies as well as their combination all reduced the tumor volume with statistically significant differences from the no-drug control tumors (*p* < 0.05–0.0001; Figure 4A, left). There was also a significant difference between the mice with cetuximab alone and with erdafitinib/cetuximab (*p* < 0.05); we found no statistically significant difference between erdafitinib alone and erdafitinib/cetuximab. These results suggested that erdafitinib played a dominant role in the therapeutic effect on the HC6T PDSXs. On the other hand, in PDSX mice of HC20T which also demonstrated in vitro sensitivity to erdafitinib, both monotherapies and their combination suppressed tumor growth at similar extents compared with the control (*p* < 0.05; Figure 4B, left). Consistently, the proportion of Ki67-positive cells in tumor sections was reduced significantly in the combination therapy groups for both spheroid lines, although no discernible changes were found in the histopathological features of PDSX tumors at the end of treatment for three weeks (*p* < 0.05; Figure 4A,B, right, Figure S3A,B).

The tumor volume relative to the initiation point of the drug dosing levelled off with time in HC6T PDSXs seemingly because after the initial growth, some of the tumor cells started to disappear by apoptosis, which was balanced by growth of new tumor cells. This interpretation was supported by many cells in the tumor stained positively with Ki67 at necropsy (Figure S3A, right).

In addition, we tested PDSX chemosensitivity of the HC1T tumor of which spheroids did not respond to either erdafitinib or erlotinib in vitro but did respond to the combination significantly (Figure 1D, Figure 2B). Although erdafitinib alone did not reduce the tumor volume significantly, both cetuximab monotherapy and erdafitinib/cetuximab combination inhibited the tumor growth with statistically significant differences from that of the control tumors (*p* < 0.01–0.0001; Figure 4C, left). There was also a significant difference between erdafitinib alone and erdafitinib/cetuximab combination (*p* < 0.05). Consistent with these results, the proportion of Ki67-positive cells was reduced significantly in the combination therapy group compared with the control or erdafitinib group (*p* < 0.05; Figure 4C, right). These results show that the combined efficacy of FGFR and EGFR inhibitors in xenografts continues beyond experiments in vitro, suggesting efficacious results in patients as well.

### 2.6. Little Association between FGFR Inhibitor Sensitivity and FGFR Gene Alterations, or mRNA Levels in Colorectal Cancer Spheroids

**Figure 5:**
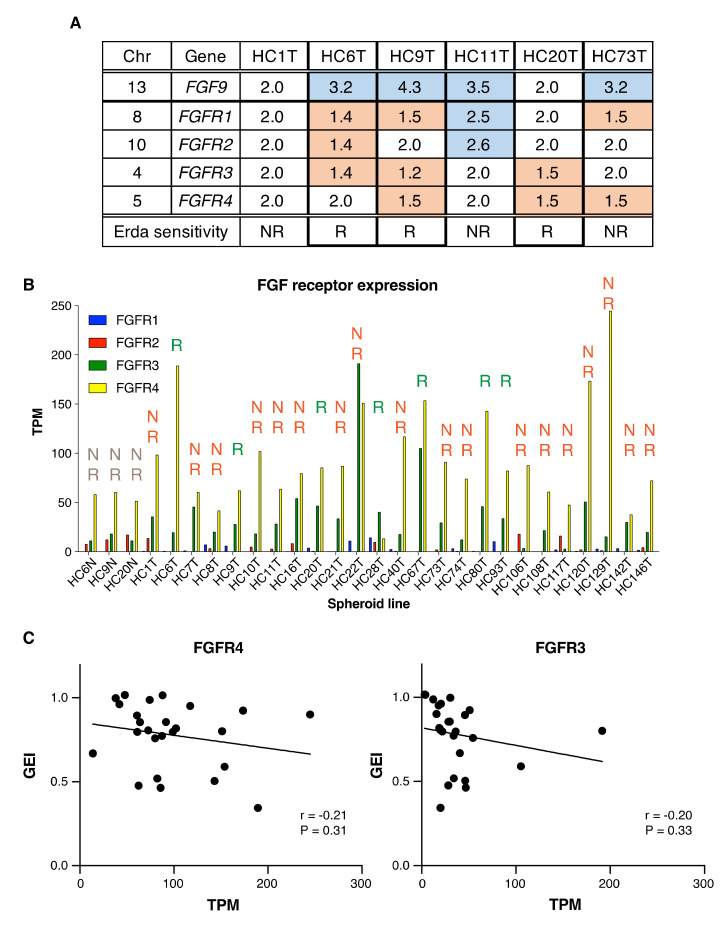
Little association between FGFR inhibitor sensitivity and *FGFR* gene alterations, or mRNA levels in colorectal cancer spheroids. (**A**), Copy numbers of the *FGF9*, and *FGFR1–4* gene loci in six colon cancer TIC-spheroid lines (HC1T, HC6T, HC9T, H11T, HC20T, and HC73T). Gain (blue) or loss (red) of the chromosomal region was determined relative to the normal epithelial genomic DNA (2.0). “R” stands for “responsive” and “NR” for “non-responsive” to erdafitinib in culture. (**B**) The *FGFR* mRNA expression levels in all 25 TIC-spheroid lines and three normal colonic epithelial stem cell lines (HC6N, HC9N, and HC20N) as determined by RNA-seq analysis (see Materials and Methods). TPM; transcripts per million in the RNA-seq samples. (**C**) Association between GEI and TPM in 25 colorectal cancer spheroid lines. There was a weak tendency between sensitivity to erdafitinib and *FGFR3/4* mRNA expression (*r* = −0.21, *r* = −0.20, respectively).

Several sets of preclinical data, based on various types of cancer cell lines or xenografts, suggested a correlation between *FGFR* gene amplification and FGFR inhibitor sensitivity [5,6,7,8]. So that we could predict the sensitivity of spheroids to FGFR inhibitors based on the *FGFR* genetic/genomic status, we analyzed the gene copy numbers for the four paralogs using comparative genomic hybridization. We found a small copy number gain in HC11T for *FGFR1* and *FGFR2* genes (2.5 and 2.6, respectively, over 2.0; Figure 5A) despite that the spheroid line was insensitive to FGFR inhibitors in vitro at pharmacological concentrations (Figure 1D and Figure 2B). In HC6T, HC9T or HC20T, on the other hand, the *FGFR1–4* gene copy numbers were neither increased nor decreased in spite of that they were all sensitive to FGFR inhibitors (Figure 1A–C, and Figure 5A). Thus, there was no correlation between the copy numbers and sensitivity to the FGFR inhibitors.

In addition, we estimated the levels of mRNA by RNA-seq analysis for all 22 *FGF* ligands and *FGFR1–4* for all 25 TIC spheroid lines as well as three normal colonic epithelial spheroid lines (HC6N, HC9N, and HC20N) (Figure 5B, C, Figure S4). As described above regarding Western analysis, *FGFR1* and *FGFR2* were hardly detected. Although mRNA for several *FGF* ligands or receptors was overexpressed in some spheroid lines, there was little correlation between the signal intensity and the sensitivity to FGFR inhibitors (Figure S4, Figure 5B,C, Figure 1A–D, and Figure S1B). For *FGFR3* and *FGFR4*, we also searched for possible association between the sensitivity to FGFR inhibitors and mRNA expression in all 25 spheroid lines (Figure 5C). There was a weak correlation between the sensitivity to erdafitinib and the levels of *FGFR3/4* mRNA (*r* = −0.21/−0.20). Regarding FGF ligands, we only found a moderate correlation between the sensitivity to erdafitinib and the mRNA level of *FGF19* (*r* = −0.35), and this could be caused by only one high expression sample (HC80T; Figure S4). We also determined the mRNA levels for *FGF* receptors and ligands in the frozen whole tumor tissues. Whereas the receptor mRNA was expressed at similar levels between the spheroids and the whole tissues, ligand mRNA was hardly expressed in the tumor tissues (Figure S5).

Like previous studies [33,34], our RNA-seq data indicated no obvious signs of translocations involving the *FGFR* genes (Figure S6A,B). As shown in Figure 5B, RNA-seq analysis revealed expression of *FGFR3/4* mRNA at levels higher than those of *FGFR1/2* in all spheroid lines tested. Notably, none of these lines had *FGFR1–4* mutations in their coding sequences (Table S1) except HC129T that contained point mutation in FGFR1 causing R820H. Furthermore, we tested 13 lines (HC1T, HC6T, HC8T, HC9T, HC13T, HC40T, HC49T, HC67T, HC73T, HC80T, HC93T, HC106T, HC146T) for the prevalence of FGFR4 G388R polymorphism [35,36], and found four heterozygotes (27%) and five homozygotes (33%). However, this polymorphism was not associated with the sensitivity to FGFR inhibitors, although it had been reported to reflect the metastatic potential and poor survival of solid tumors including colorectal cancer [35,36].

Accordingly, it is unlikely that the spheroid lines sensitive to FGFR inhibitors can be easily selected solely based on genetic/genomic data such as amplification and overexpression. Therefore, we conclude that the assessment of chemosensitivity directly on TIC spheroids is a better correlated strategy that helps select colorectal cancer patient subpopulations that can benefit from FGFR inhibitors.

## 3. Discussion

In the present study, we reported for the first time that approximately one-third of colorectal cancer patients with wild-type *RAS/RAF* may benefit from FGFR inhibitor therapy by demonstrating the chemosensitivity of patient-derived TIC spheroid lines (Table 1, Figure 1, and Figure S1); in vitro evaluation is rather quick, requiring only 1–2 months. Additionally, the dosing effects in mouse xenografts (PDSXs) of three representative lines translationally matched those of the corresponding spheroid lines in culture (Figure 4). In our previous report, we demonstrated the validity of PDSX models to predict effects of chemotherapeutic agents such as 5-FU, irinotecan, cetuximab, etc. [21]. Also, Vlachogiannis et al. [19] reported in their multiple clinical trials that the positive predictability of PDSX results from clinical patients is nearly 90%; this is much higher than genomics-based patient selections. These results strongly suggest that in vitro drug-dosing tests help optimize the selection of colorectal cancer patients who are likely to benefit from FGFR inhibitor therapy. To evaluate cancer chemosensitivity, it is much quicker to use in vitro culture of cancer spheroids than preparing whole animal xenografts such as PDXs or PDSXs, although spheroid cultures can give answers only for drugs that affect epithelial cell-autonomous proliferation. The most practical cases are high-risk Stage II and Stage III. Prognosis for Stage 0–I patients is very good without chemotherapy. For most of the Stage IV patients, primary tumors may not be available.

In most of the previous clinical trials, enrolled patients had *FGFR* gene aberration such as *FGFR* amplification and mRNA overexpression [9,10,11,12,13,14,15,16,17]. However, except urothelial cancers for which the FDA has approved erdafitinib [37], the responses to FGFR inhibitors were minimal [17]. In our study on colorectal cancer, *FGFR* gene copy numbers or expression levels did not necessarily match with their sensitivity to the FGFR inhibitors (Table 1, Figure 5). This result can explain the limited effects shown in the previous clinical trials on FGFR inhibitors.

We previously reported that growth of the four spheroids lines (HC6T, HC9T, HC11T and HC21T) were strongly accelerated by the FGF ligand [20]. As shown in the present study, however, HC6T and HC9T were sensitive to FGFR inhibitors, whereas HC11T and HC21T were resistant (Table 1). This result suggested that FGF dependency was not necessarily associated with the sensitivity to FGFR inhibitors. Furthermore, as shown in Figure S5, mRNA for FGF ligands was hardly detected in tumor tissues for the three lines sensitive to FGFR inhibitors (HC6T, HC9T, and HC20T). These results led us to speculate that autocrine secretion of ligands may be one of the important mechanisms that can affect the sensitivity to FGFR inhibitors.

Notably, we have found that combination of FGFR and EGFR inhibitors is efficacious in vitro not only for the spheroid lines sensitive to the FGFR inhibitor alone, but also for those insensitive to it (Table 1). In addition, this combination efficacy was replicated in PDSX mice in three representative spheroid lines we tested (Figure 4). These results suggest that patient-derived TIC spheroids help predict the chemosensitivity to combination regimens such as erdafitinib and cetuximab or panitumumab regarding colorectal cancer patients in the clinical setting.

It is well known that molecularly targeted therapy tends to induce specific and acquired resistance rather quickly in vitro and in vivo. Several recent clinical studies showed resistance mechanisms against FGFR inhibitors, i.e., *FGFR* gene point mutations and pathway activation such as *MET* [16,38,39,40]. For example, *EGFR* suppresses *FGFR3* expression in cells that are resistant to FGFR3 inhibitors and dominates the signal input to downstream pathways [41]. Accordingly, we anticipate that the efficacy on the primary tumor cells may be retained in the metastatic lesions until specific resistant subclones dominate the tumor [42]. Prospective clinical studies should help resolve such problems in more practical terms based on the results of in vitro chemosensitivity studies of TIC spheroids combined with the monitoring of resistant subclones through cell-free DNA [38,40].

In chemotherapy of colorectal cancer, the therapeutic efficacy is usually as low as 50% [43,44]. This indicates that a sizable fraction of patients who receive these therapies does not respond favorably, and therefore lose their precious time and quality-of-life with unmet expectations. Thus, it is important not only to select patients who can benefit from the chemotherapy regimens, but also to exclude patients who cannot. Even if there are extra costs to establish the spheroid cultures and test in vitro or with xenografts, such costs will be well offset by the expensive costs of chemotherapy. In addition, primary tumor specimens are usually available from most colorectal cancer patients at Stages II and III, and culturing spheroids in vitro imposes no physical or psychological burden to patients. With the improved efficiency of spheroid establishment at near 90% [20], this ‘para-clinical’ approach can become one of the standard companion diagnostic methods for colorectal cancer chemotherapy.

## 4. Materials and Methods

### 4.1. Human Samples

Human colorectal cancer samples were obtained from patients who underwent resection operations at Department of Surgery, Kyoto University Hospital from 2014 to 2017. The study protocol was approved by the Ethics Committee of Kyoto University, and written informed consents were obtained from all patients (Approval No. R0915 and R0857, FY2015–2019).

### 4.2. Patient-Derived Cancer-Spheroid Culture

The procedures were described previously for establishing primary cancer spheroids and normal epithelial spheroids from tumor samples [20,27]. In short, collected tumor samples were transferred from the operation room to the laboratory in ice-cold washing medium (DMEM/F12 containing 10% bovine calf serum, 100 U/mL penicillin, and 0.1 mg/mL streptomycin). Fragments of excised tumor were minced and digested by collagenase type I (Thermo Fisher Scientific, Waltham, MA, USA). Then, epithelial cells were collected and suspended in Matrigel (Corning Inc., Corning, NY) and cultured in the cancer medium [20] with or without 50 ng/mL EGF (PeproTech, Cranbury, NJ) and 100 ng/mL basic FGF (PeproTech).

### 4.3. Immunoblotting Analysis

Spheroids were treated with DMSO or drugs in DMSO for 2 h, harvested and lysed as described previously [45]. Aliquots (5 µg-protein each) were used to perform analysis by SDS-PAGE, electro-transfer and immunoblotting with the antibodies for the following; pFGFR Tyr653/654, FGFR4, FGFR3, pEGFR Tyr1068, EGFR, pMEK1/2 Ser217/221, MEK1/2, pERK1/2 Thr202/Tyr204 and ERK1/2 (1:1000 dilution), all from Cell Signaling Technology, Danvers, MA, USA, and ACTB (1:20,000 dilution), Sigma–Aldrich, St. Louis, MO.

### 4.4. Chemicals

Erdafitinib (JNJ42756493, pan-FGFR1–4 inhibitor; Active Biochem, Kowloon, Hong Kong), AZD4547 (FGFR1–3 inhibitor; Active Biochem), infigratinib (BGJ398, FGFR1–3 inhibitor; AdooQ, Irvine, CA, USA), fisogatinib (BLU554, FGFR4-selective inhibitor; Selleck Chemicals, Houston, TX), rogaratinib (BAY1163877, FGFR1–3 inhibitor; MedChemo Express, Monmouth Junction, NJ), LY2874455 (Chem Scene, pan-FGFR1–4 inhibitor; Monmouth Junction, NJ), TAS-120 (pan-FGFR1–4 inhibitor; Cayman Chemical Company, Ann Arbor, MI), and erlotinib (EGFR inhibitor; Chem Scene) were dissolved and diluted in DMSO. Cetuximab (Erbitux, anti-EGFR antibody, Merck, Kenilworth, NJ) was diluted in PBS.

### 4.5. Luminescence-Based Drug-Dosing Tests in Spheroid Culture

Luciferase-expressing spheroids were cultured using 96-well “white” cell-culture plates (Corning; 3 μL per well). The luminescence scoring system was described previously [20]. Luminescence was determined on day 1 and 4, and the effects of drug dosing were evaluated by computation of GEI (growth effect index; defined as the growth rate of a treated group relative to that of its solvent-only control) in each paired assay. The cut-off GEI between “responsive” and “non-responsive” was assigned at 0.7 (70%).

### 4.6. Preparation of Spheroid DNA and RNA Samples

DNA; Spheroids in Matrigel were suspended in Cell Recovery Solution (Corning), and collected in 1.5 mL tubes. Matrigel was digested at 4 °C with rotation for 30 min. Spheroids were centrifuged for 5 min and washed with PBS twice at 4 °C. DNA was purified using DNeasy Blood & Tissue Kit (Qiagen, Hilden, Germany).

RNA; After aspiration of the medium, the lysis buffer (Takara Bio, Kusatsu, Japan) was directly added to each well of spheroid culture. RNA was purified using NucleoSpin RNA II kit (Takara Bio).

### 4.7. Array-Based Comparative Genomic Hybridization (CGH) Analysis

Array-based CGH analyses were performed using Agilent SurePrint G3 human CGH microarray 1 × 1 M (Agilent, Santa Clara, CA, USA) by Macrogen (Seoul, Republic of Korea). Human Genomic DNA (G1521 and G1471, Promega, Madison, WI) was used as the reference.

### 4.8. Mutation Analysis

The mutational status is shown in Table 1 and Table S1 for each spheroid sample. Targeted next-generation sequencing of cancer-related genes were performed by Macrogen. In brief, amplicon libraries were prepared using Ion AmpliSeq Comprehensive Cancer Panel (Thermo Fisher), and sequenced with the Ion Proton sequencer (Thermo Fisher). The sequencing data were processed using Ion Torrent Suite Software v5.0.4 (Thermo Fisher), and variants against the hg19 human genome reference were called using Torrent Variant Caller v5.0.4 (Thermo Fisher).

### 4.9. Filtration of Variants

The called variants were annotated using ANNOVAR software [46] and filtered to select nonsynonymous, frameshift, and splicing mutations with >20% frequency. Then, the polymorphic alleles were removed by referring to two databases; Human Genetic Variation Database (HGVD) and 1KJPN [47,48]. Erroneous mutations were also eliminated by surveying their coverage tracks on Integrative Genomics Viewer software v2.3 (Broad Institute, Cambridge, MA, USA).

### 4.10. RNA-Seq Analysis

RNA-seq analysis was performed by Macrogen. In brief, prepared libraries were paired-end sequenced (2 × 100 bases) to a depth of ~40 million reads/sample using the Illumina HighSeq 2000 sequencer. The sequence reads were mapped to the hg19 reference genome with HISAT2 program [49] and the transcripts were assembled using StringTie program [50]. The read counts were scored using featureCounts program [51].

### 4.11. PDSX (Patient-Derived Spheroid Xenograft)

Four to five-week-old female nude (BALB/c-*nu*) mice were purchased from Charles River (Wilmington, MA, USA). All animal experiments were conducted according to the protocol approved by the Animal Care and Use Committee of Kyoto University (IACUC): Title of the protocol, “Chemosensitivity studies of gastrointestinal cancers using patient-derived tumor xenografts.” Approval No. 14546, 15091, 16047, 16654, 17086, 18080, and 19601 (2014–2019).

### 4.12. Drug Sensitivity Tests in Mice

PDSX mice were prepared and subjected to drug-dosing test when the mean estimated tumor volume reached ~300 mm^3^. Tumor volume (in mm^3^) was estimated by the following formula: length × width^2^/2. We excluded the following kinds of tumors from the tests as outliers; too small (<100 mm^3^), spontaneously shrinking, remaining unchanged in size for more than two weeks, or deeply implanted and difficult to be measured. The mice in each dosing group were given with erdafitinib orally and/or with cetuximab intraperitoneally at clinically relevant doses for 21 days. For erdafitinib treatment, mice were dosed orally with MediGel Sucralose (ClearH_2_O, Portland, ME) containing the drug at 10 mg/mL, as reported in previous reports [6,12,15]. For cetuximab treatment, mice were injected intraperitoneally (i.p.) with 250 μg of the drug per injection twice a week for three weeks, which was calculated according to the following formula: mouse dose (mg/kg) = human dose (mg/kg) × 37 (hKm)/3 (mKm) where Km indicates human or mouse body surface coefficient [21,52,53].

The relative tumor volume was estimated by calibration to the initial tumor volume on day 0. To evaluate the drug dosing effects, the ratio of tumor volume on day 21 to that on day 0 was compared among the four groups.

### 4.13. Histological Examinations

Paraffin-embedded tissues of PDSXs were prepared according to standard procedures. Sections were stained with H&E or immunostained for Ki67.

### 4.14. Statistics

Data were analyzed using one-way or two-way ANOVA followed by Tukey’s post-test, or paired *t*-test using GraphPad Prism ver.8 (GraphPad software. Inc., San Diego, CA, USA).

## 5. Conclusions

Direct testing of the patient-derived spheroids in vitro is a robust personalized method to identify the subset of colorectal cancer patients with potential benefit from FGFR-inhibitor therapy.

## Figures and Tables

**Table 1:** Clinicopathological characteristics, mutational statuses, and responses to FGFR/EGFR inhibitors of 25 patient-derived *RAS/RAF* wild-type colorectal cancer spheroids used in the present study. FGFR: fibroblast growth factor receptor; EGFR: epidermal growth factor receptor.

ID	Patient		Tumor		Pathological Stage	Mutations in Representative Cancer-Related Genes				Drug Sensitivity ^c^ (GEI)					
	Age	Sex	Location ^a^	Histology ^b^						Erdafitinib		Erlotinib		Erdafitinib/Erlotinib	
										(FGFR Inhibitor)		(EGFR Inhibitor)		Combination	
HC6T	57	M	S	Well-Mod	4	*APC*	*TP53*			R (0.34)			NR	**R** (0.25)	
HC9T	73	M	A	Well-Mod	2	*APC*	*TP53*			R (0.47)			NR	**R** (0.20)	
HC20T	66	F	S	Well-Mod	4		*TP53,*			R (0.43)			NR	**R** (0.24)	
HC28T	71	M	D	Well-Mod	2	*APC*		*PIK3CA*		R (0.66)			NR	**R** (0.25)	
HC67T	72	M	R	Well-Mod	2	*APC*				R (0.58)			NR	**R** (0.28)	
HC80T	75	M	S	Well-Mod	4	*APC*	*TP53*			R (0.50)			NR	**R** (0.32)	
HC93T	66	F	R	Well-Mod	3		*TP53*			R (0.51)			NR	**R** (0.28)	
HC7T	68	F	A	Well-Mod	2		*TP53*				NR	R (0.43)		**R** (0.30)	
HC10T	70	F	C	Well-Mod	2	*APC*	*TP53*				NR	R (0.67)		**R** (0.30)	
HC73T	75	M	S	Well-Mod	4	*APC*	*TP53*				NR	R (0.65)		**R** (0.39)	
HC108T	42	F	R	Well-Mod	4	*APC*	*TP53*				NR	R (0.57)		**R** (0.31)	
HC117T	77	M	S	Mucinous	3	*APC*	*TP53*				NR	R (0.62)		**R** (0.20)	
HC1T	81	F	A	Well-Mod	4	*APC*		*PIK3CA*			NR		NR	**R** (0.46)	
HC8T	66	F	T	Well-Mod	1	*APC*		*PIK3CA*	*FBXW7*		NR		NR	**R** (0.31)	
HC11T	74	M	T	Mucinous	3	*APC*	*TP53*				NR		NR	**R** (0.41)	
HC16T	89	M	R	Well-Mod	3	*APC*	*TP53*		*FBXW7*		NR		NR	**R** (0.57)	
HC21T	52	M	R	Well-Mod	4	*APC*	*TP53*		*FBXW7*		NR		NR	**R** (0.35)	
HC22T	51	M	S	Well-Mod	2	*APC*	*TP53*				NR		NR	**R** (0.58)	
HC74T	50	M	S	Well-Mod	4	*APC*	*TP53*				NR		NR	**R** (0.67)	
HC142T	67	F	R	Well-Mod	4						NR		NR	**R** (0.34)	
HC146T	45	F	R	Well-Mod	4		*TP53*				NR		NR	**R** (0.36)	
HC40T	82	M	T	Well-Mod	3	*APC*	*TP53*	*PIK3CA*			NR		NR		NR
HC106T	63	F	T	Well-Mod	1	*APC*					NR		NR		NR
HC120T	55	M	S	Well-Mod	3		*TP53*				NR		NR		NR
HC129T	55	M	R	Well-Mod	2	*APC*	*TP53*				NR		NR		NR
Sensitivity										7 (28%)		5 (20%)		21 (84%)	

^a^ C: cecum; A, T, D, and S: ascending, transverse, descending, and sigmoid colon, respectively, R: rectum; ^b^ Well-Mod, well-to-moderately differentiated; ^c^ R, responsive; **R** (bold face), more responsive than R in monotherapy; NR, non-responsive, i.e., GEI > 0.7, (GEI; the growth rates of treated spheroids relative to those with solvent control, see Materials and Methods, Section 4).

## Supplementary Materials

The following are available online at https://www.mdpi.com/2072-6694/12/8/2010/s1, Figure S1: Effects of erlotinib or cetuximab on the growth of three colon cancer spheroid lines, HC6T, HC9T, and HC20T. Figure S2: Growth of PDSX tumors derived from colorectal cancer TIC spheroid lines before drug treatments. Figure S3: Histological specimens of xenograft tumors derived from HC6T, HC20T, and HC1T spheroid lines. Figure S4: The levels of mRNA for all 22 *FGF* ligands in the 25 spheroid lines. Figure S5: The mRNA levels for *FGF* receptors and the ligands in frozen whole tumor tissues for HC6T, HC9T, and HC20T determined by RNA-seq analysis. Figure S6: Sashimi plots across RNA-seq samples of *FGFR4/FGFR3* mRNA in all 25 colorectal cancer TIC-spheroid lines. Figure S7: Uncropped Western Blot images. Table S1: Mutational status detected by targeted next-generation sequencing of 409 cancer-related genes in each spheroid sample. Table S2: List of 409 cancer-related genes investigated in the present study.
