# Supplementary material for: Chemosensitivity of Patient-Derived Cancer Stem Cells Identifies Colorectal Cancer Patients with Potential Benefit from FGFR Inhibitor Therapy
Source: Cancers (Basel). 2020 Jul 22;12(8):2010. doi: 10.3390/cancers12082010 (PMC7465102; doi:10.3390/cancers12082010)
Supplement: Supplementary file 1 [file cancers-12-02010-s001.pdf]

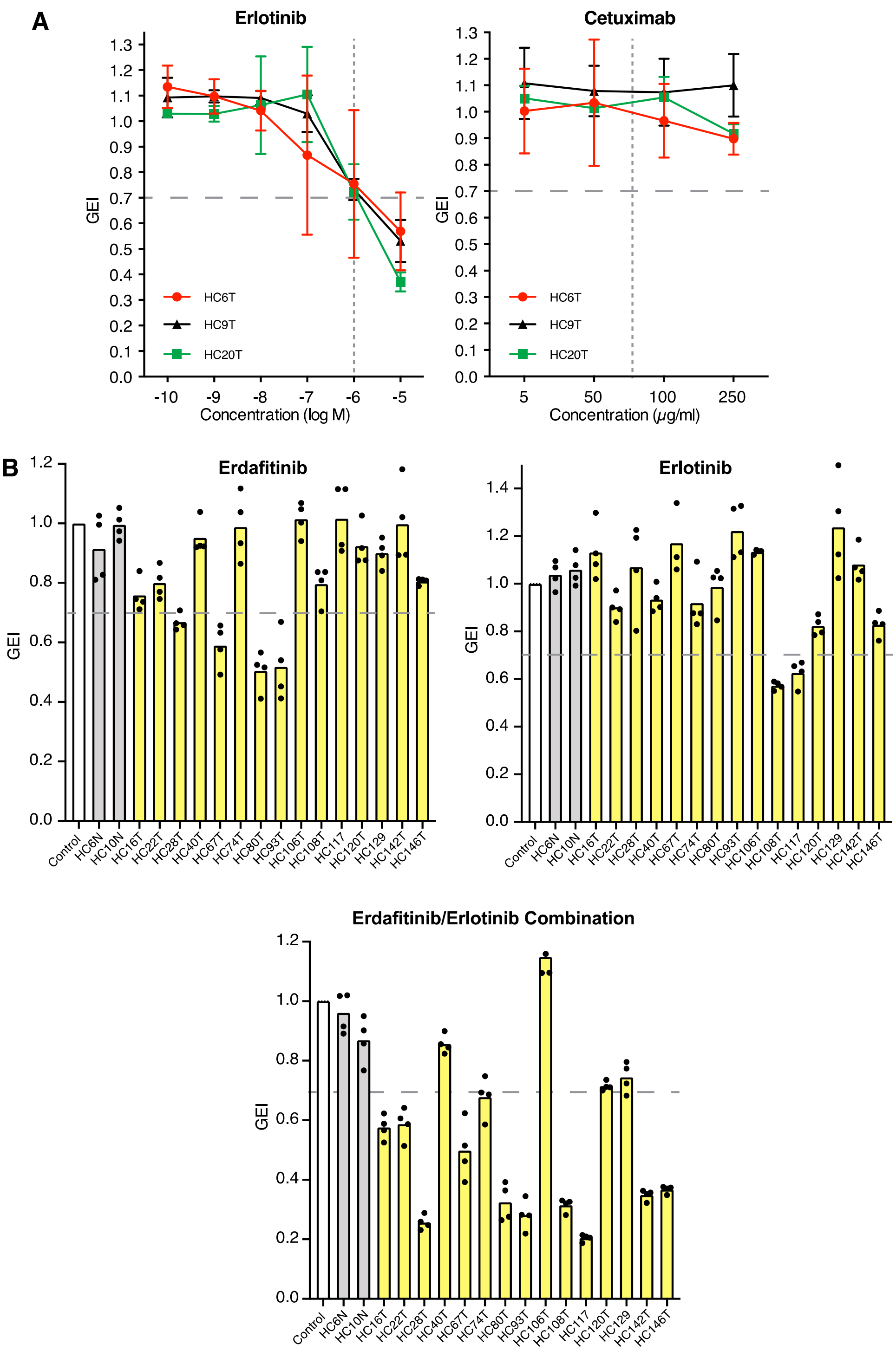

Figure S1. Effects of erlotinib (**A**. left) or cetuximab (**A**. right) on the growth of three colon cancer spheroid lines, HC6T, HC9T, and HC20T. Dose-dependent growth inhibition was observed by erlotinib but not by cetuximab on all three spheroid lines in vitro likely because of its poor permeability to the culture medium that contained Matrigel. GEI ~0.7, efficacy threshold, dashed lines; clinical doses, 10^{-6} M in erlotinib and ~80 μg/mL in cetuximab, dotted lines. Effects of erdafitinib (0.1 μM), erlotinib (1 μM), or erdafitinib (0.1 μM)/erlotinib (1 μM) combination on the in vitro growth of 15 *RAS*/*RAF*-wild type colorectal cancer TIC spheroid lines and two normal colorectal epithelial stem cell spheroid lines (HC6N, HC10N) (**B**). The mean values of four replicates are shown as bars with raw data points as dots. The cut-off value of GEI that classifies spheroids as "responsive" or "non-responsive" was assigned at 0.7 (broken line).

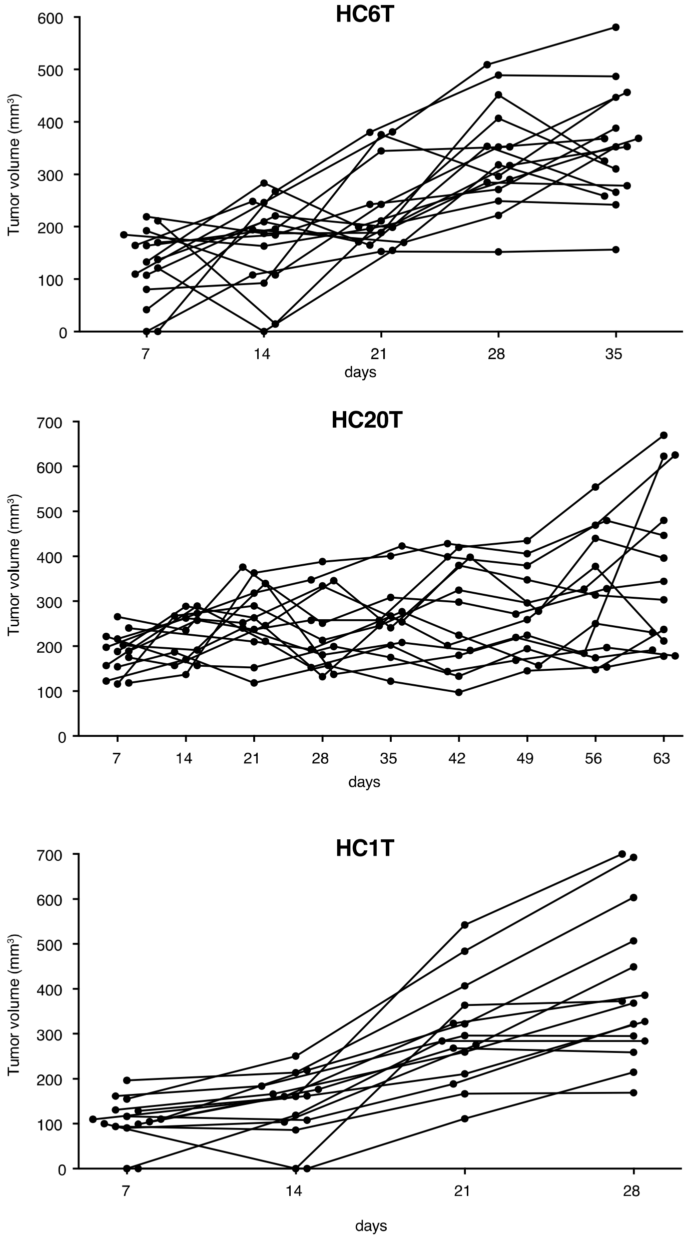

Figure S2. Growth of PDSX tumors derived from colorectal cancer TIC spheroid lines (top, HC6T; middle, HC20T; and bottom, HC1T) before drug treatments. Mice with xenografts of the following characteristics were excluded from the dosing tests; too small (<100 mm^3), spontaneously shrinking, remaining unchanged in size for more than two weeks, or deeply implanted and difficult to be measured.

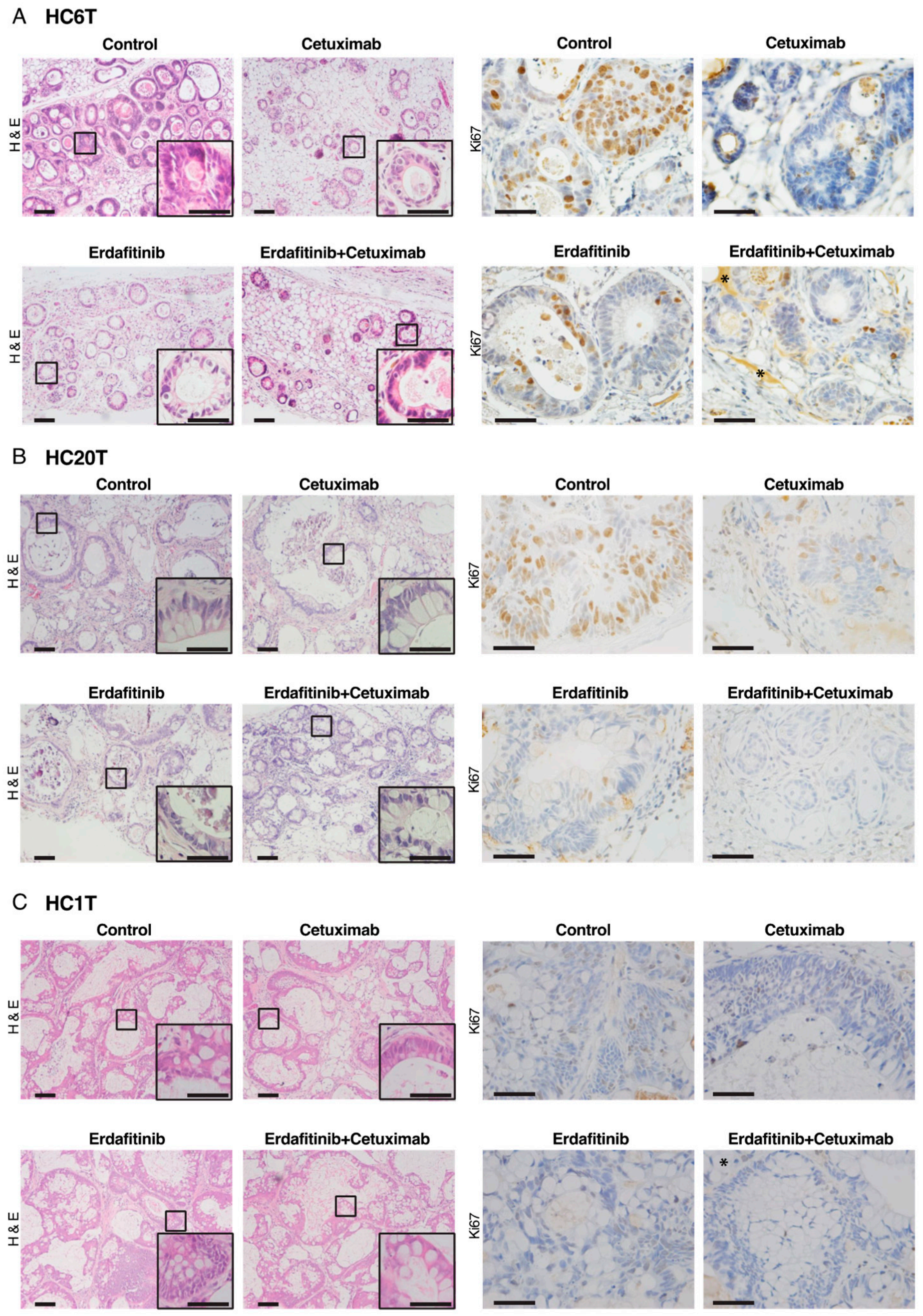

Figure S3. Histological specimens of xenograft tumors derived from HC6T (**A**), HC20T (**B**), and HC1T (**C**) spheroid lines on day 21 stained with H&E (left two columns) or immunostained for a proliferation marker, Ki67 (right two columns). Magnification bars, 100 µm or 50 µm (insets).

	FGF1	*FGF2*	*FGF3*	*FGF4*	*FGF5*	*FGF6*	*FGF7*	*FGF8*	*FGF9*	*FGF10*	*FGF11*	*FGF12*	*FGF13*	*FGF14*	*FGF16*	*FGF17*	*FGF18*	*FGF19*	*FGF20*	*FGF21*	*FGF22*	*FGF23*	Erda	Erlo	Erda/Erlo
HC6N	0.00	0.90	0.00	0.00	0.00	0.00	0.00	0.19	0.00	0.00	0.95	0.00	0.03	0.00	0.00	0.22	0.00	0.29	0.00	3.78	0.00	0.03			
HC9N	0.00	3.32	0.00	0.00	0.00	0.00	0.00	0.00	0.00	0.00	9.35	0.00	0.00	0.00	0.00	0.00	0.22	0.82	0.00	0.00	0.00	0.00			
HC20N	0.00	1.31	0.00	0.00	0.00	0.00	0.00	0.00	0.00	0.00	4.02	0.00	0.00	0.00	0.00	0.00	0.04	0.11	0.00	0.00	0.18	0.00			
HC6T	0.00	0.02	10.12	0.36	0.00	0.00	0.00	0.00	0.39	0.00	1.80	0.00	0.25	0.00	0.00	0.37	6.87	38.30	40.06	0.00	0.23	0.05	**R**	**NR**	**R**
HC9T	0.03	0.00	1.09	0.00	0.00	0.00	0.00	0.00	0.09	0.00	1.41	0.06	0.04	0.00	0.00	0.19	8.44	0.67	4.60	0.00	0.81	0.00	**R**	**NR**	**R**
HC20T	0.05	0.00	0.25	0.00	0.00	0.00	0.00	0.23	0.20	0.00	1.88	0.00	0.04	0.00	0.00	0.00	0.39	2.34	23.96	0.00	0.80	0.13	**R**	**NR**	**R**
HC28T	0.05	3.48	51.10	0.00	0.00	0.00	0.00	0.89	6.64	0.00	0.70	0.00	0.14	0.00	0.00	0.89	6.64	52.02	7.39	0.14	0.57	0.00	**R**	**NR**	**R**
HC67T	0.06	0.00	0.20	0.00	0.00	0.00	0.04	0.00	2.55	0.00	1.90	0.00	0.18	0.00	0.00	0.00	3.35	1.04	29.25	0.36	0.00	0.16	**R**	**NR**	**R**
HC80T	0.02	0.00	9.23	0.00	0.00	0.00	0.00	1.58	0.49	0.00	2.27	0.02	0.03	0.00	0.00	0.25	2.01	176.46	15.79	0.13	0.36	0.47	**R**	**NR**	**R**
HC93T	0.06	0.00	1.12	1.42	0.00	0.00	0.00	0.52	10.19	0.00	0.51	0.00	0.13	0.00	0.00	0.31	5.24	17.01	5.54	0.00	1.79	0.05	**R**	**NR**	**R**
HC7T	0.06	0.00	0.21	0.00	0.00	0.00	0.00	0.00	0.43	0.00	1.17	0.02	0.22	0.00	0.00	0.08	9.25	2.78	35.70	0.12	0.00	0.40	**NR**	**R**	**R**
HC10T	0.00	0.30	0.00	0.00	0.00	0.00	0.00	0.13	0.00	0.00	0.71	0.00	0.00	0.00	0.00	0.00	0.36	0.74	0.00	0.65	0.00	0.00	**NR**	**R**	**R**
HC73T	0.00	0.00	0.13	0.00	0.00	0.00	0.00	0.09	0.00	0.00	21.13	0.02	0.39	0.00	0.00	0.30	0.42	0.87	0.10	0.23	0.00	0.00	**NR**	**R**	**R**
HC108T	0.00	0.00	1.97	0.00	0.00	0.00	0.00	0.00	1.51	0.00	1.90	0.02	0.08	0.00	0.00	0.00	10.09	11.31	34.46	1.57	0.00	1.01	**NR**	**R**	**R**
HC117T	0.00	1.50	0.10	0.00	0.00	0.00	0.00	0.27	0.07	0.00	0.54	0.05	0.00	0.05	0.00	0.11	1.19	0.31	0.15	0.00	0.23	0.00	**NR**	**R**	**R**
HC1T	0.00	0.16	0.00	0.00	0.00	0.18	0.00	0.00	0.18	0.00	2.35	0.00	0.00	0.00	0.00	0.10	0.14	0.00	0.13	0.00	0.21	0.04	**NR**	**NR**	**R**
HC8T	0.00	2.24	0.00	0.00	0.00	0.00	0.00	0.88	0.25	0.00	1.01	0.09	0.04	0.05	0.00	0.10	2.30	3.41	35.60	0.00	1.09	0.09	**NR**	**NR**	**R**
HC11T	0.00	0.04	0.09	0.00	0.00	0.00	0.00	0.12	0.00	0.00	2.07	0.02	0.16	0.00	0.00	0.10	0.21	0.51	0.00	0.00	0.21	0.00	**NR**	**NR**	**R**
HC16T	0.00	0.09	0.56	0.00	0.00	0.00	0.00	0.00	0.29	0.00	0.89	0.00	0.21	0.00	0.00	0.00	1.39	2.10	0.57	0.00	0.11	0.19	**NR**	**NR**	**R**
HC21T	0.00	0.29	0.09	0.00	0.00	0.00	0.00	0.00	0.32	0.00	1.08	0.02	0.12	0.00	0.00	0.00	2.69	5.87	10.64	0.00	0.00	0.00	**NR**	**NR**	**R**
HC22T	0.06	0.02	27.24	7.73	0.03	0.00	0.00	33.85	38.32	0.00	1.73	0.55	0.16	0.00	0.00	1.05	10.76	21.14	82.64	0.00	1.67	0.00	**NR**	**NR**	**R**
HC74T	0.00	0.04	1.12	0.00	0.00	0.14	0.00	0.09	4.09	0.00	0.46	0.00	0.18	0.00	0.00	0.07	1.10	1.16	0.25	0.23	0.08	0.00	**NR**	**NR**	**R**
HC142T	0.11	1.00	0.06	0.00	0.09	0.00	0.50	0.08	0.18	0.00	1.46	0.01	0.49	0.09	0.00	0.13	13.32	1.14	27.79	0.15	0.21	0.22	**NR**	**NR**	**R**
HC146T	0.00	0.04	1.51	0.00	0.00	0.00	0.00	0.12	0.09	0.00	1.07	0.00	0.41	0.00	0.00	0.66	0.13	3.77	0.00	0.00	0.00	0.09	**NR**	**NR**	**R**
HC40T	0.00	0.04	7.28	0.00	0.00	0.00	0.00	0.00	5.21	0.00	0.95	0.00	0.28	0.00	0.00	0.09	7.07	6.84	11.08	0.00	0.56	0.00	**NR**	**NR**	**NR**
HC106T	0.00	0.35	1.72	0.00	0.00	0.00	0.00	0.27	5.58	0.00	1.82	0.02	0.00	0.24	0.00	0.21	7.09	49.62	2.91	0.00	0.00	0.00	**NR**	**NR**	**NR**
HC120T	0.00	0.00	0.27	0.00	0.00	0.00	0.00	0.12	0.18	0.00	0.57	0.02	0.00	0.00	0.00	0.00	5.74	3.45	0.20	0.00	0.43	0.00	**NR**	**NR**	**NR**
HC129T	0.06	0.40	0.85	0.09	0.00	0.00	0.00	0.00	0.21	0.00	3.49	0.05	0.23	0.00	0.00	0.12	2.41	2.61	2.24	0.13	0.18	0.53	**NR**	**NR**	**NR**
r value	**-0.16**	**0.19**	**-0.20**	**-0.05**	**0.23**	**0.15**	**0.22**	**0.01**	**0.00**		**0.05**	**0.08**	**0.12**	**0.40**		**-0.17**	**0.02**	**-0.35**	**-0.18**	**0.04**	**-0.29**	**-0.03**			

Figure S4. The levels of mRNA for all 22 *FGF* ligands in the 25 spheroid lines. The association (*r*-value) between FGFR inhibitor sensitivity (GEI) and mRNA levels (TPM) is shown at the bottom column. The background color shows the level of expression ranging from blue (for the highest) to red (for the lowest). There was a correlation of the sensitivity to erdafitinib with the mRNA level of *FGF19* (*r* = -0.35), but not with those of other *FGF* ligands. Abbreviations R and NR stand for "responsive" and "non-responsive", respectively, to erdafitinib, erlotinib, and the erdafitinib/erlotinib combination. Note that no human *FGF15* is assigned whereas *Fgf15* is the mouse ortholog of human *FGF19*.

A

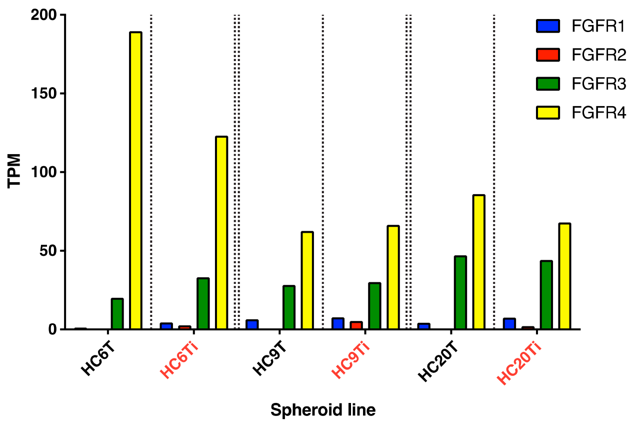

B

	FGF1	*FGF2*	*FGF3*	*FGF4*	*FGF5*	*FGF6*	*FGF7*	*FGF8*	*FGF9*	*FGF10*	*FGF11*	*FGF12*	*FGF13*	*FGF14*	*FGF16*	*FGF17*	*FGF18*	*FGF19*	*FGF20*	*FGF21*	*FGF22*	*FGF23*
HC6T	0.00	0.02	10.12	0.36	0.00	0.00	0.00	0.00	0.39	0.00	1.80	0.00	0.25	0.00	0.00	0.37	6.87	38.30	40.06	0.00	0.23	0.05
HC6Ti	0.27	0.47	1.32	0.00	0.00	0.00	1.54	0.00	0.09	0.22	2.05	0.03	2.30	0.04	0.00	0.29	3.08	0.67	3.61	0.00	0.00	0.00
HC9T	0.03	0.00	1.09	0.00	0.00	0.00	0.00	0.00	0.09	0.00	1.41	0.06	0.04	0.00	0.00	0.19	8.44	0.67	4.60	0.00	0.81	0.00
HC9Ti	0.42	2.34	0.32	0.00	0.00	0.00	5.02	0.00	0.77	0.52	1.31	0.08	1.16	0.05	0.00	0.00	3.65	0.76	0.80	0.00	0.00	1.19
HC20T	0.05	0.00	0.25	0.00	0.00	0.00	0.00	0.23	0.20	0.00	1.88	0.00	0.04	0.00	0.00	0.00	0.39	2.34	23.96	0.00	0.80	0.13
HC20Ti	0.09	0.15	0.09	0.00	0.00	0.00	1.02	0.00	0.42	0.44	0.58	0.00	0.06	0.00	0.00	0.00	0.38	0.00	0.00	0.00	0.00	0.00

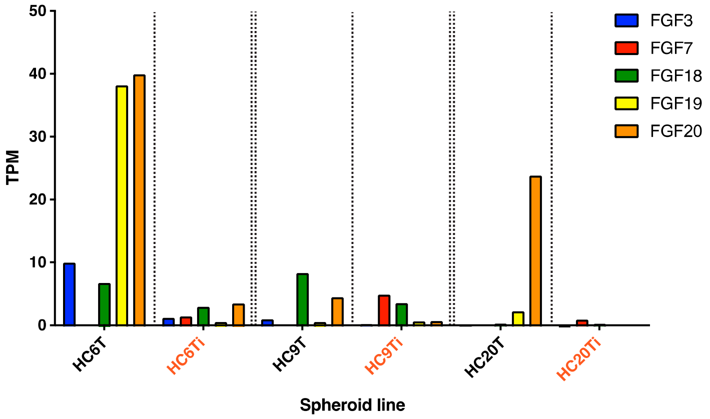

Figure S5. The mRNA levels for *FGF* receptors (**A**) and the ligands (**B**) in frozen whole tumor tissues for HC6T, HC9T, and HC20T determined by RNA-seq analysis (in transcripts per million; TPM). Note that the *FGFR* mRNA levels were similar between the tumor tissues (HC6Ti, HC9Ti, and HC20Ti) and corresponding spheroid lines (HC6T, HC9T, and HC20T) (**A**), whereas the ligand mRNA levels were relatively low in all the three tested whole tissues to the corresponding spheroid lines (**B**).

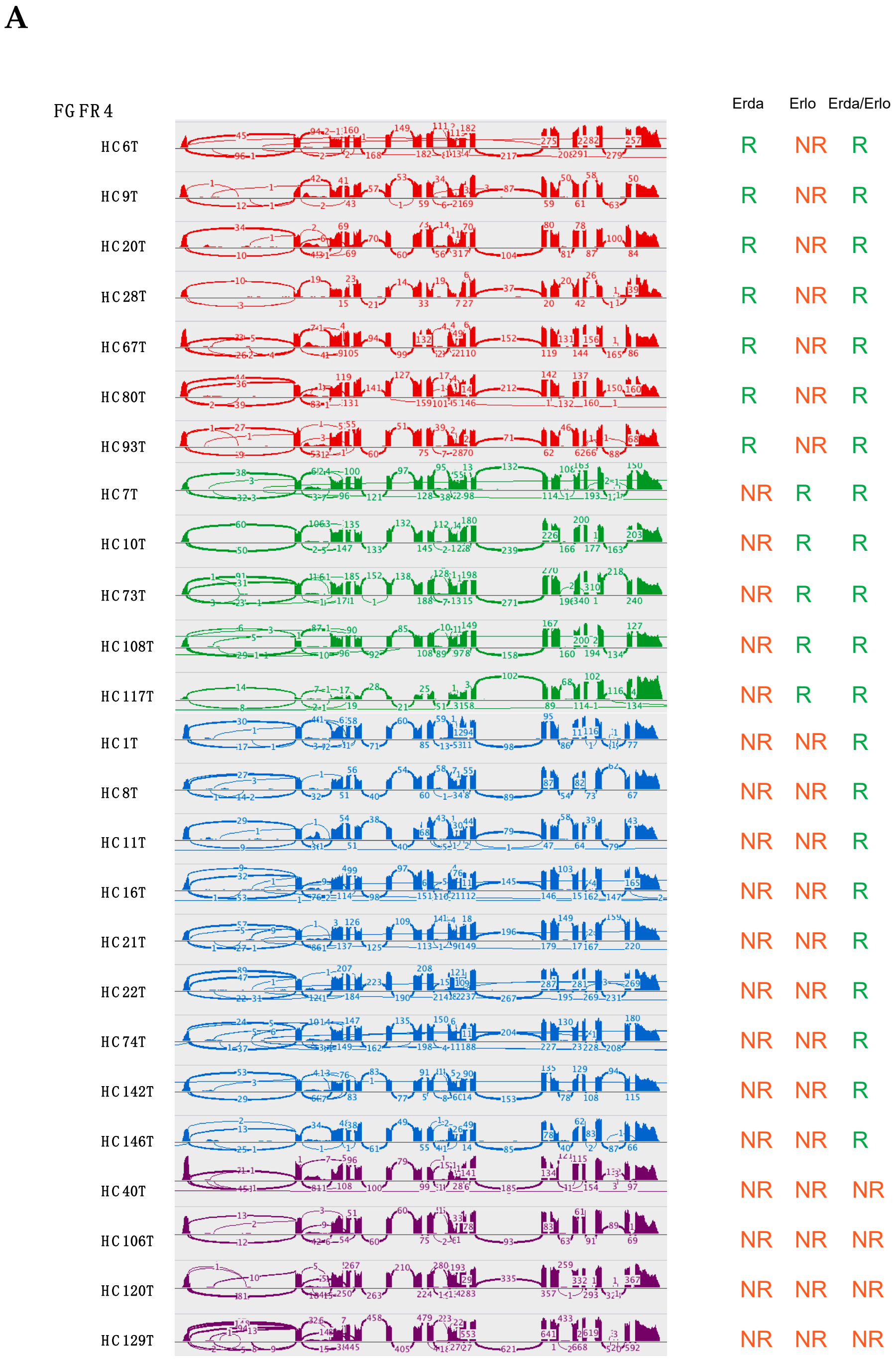

Figure 6. *Cont.*

B

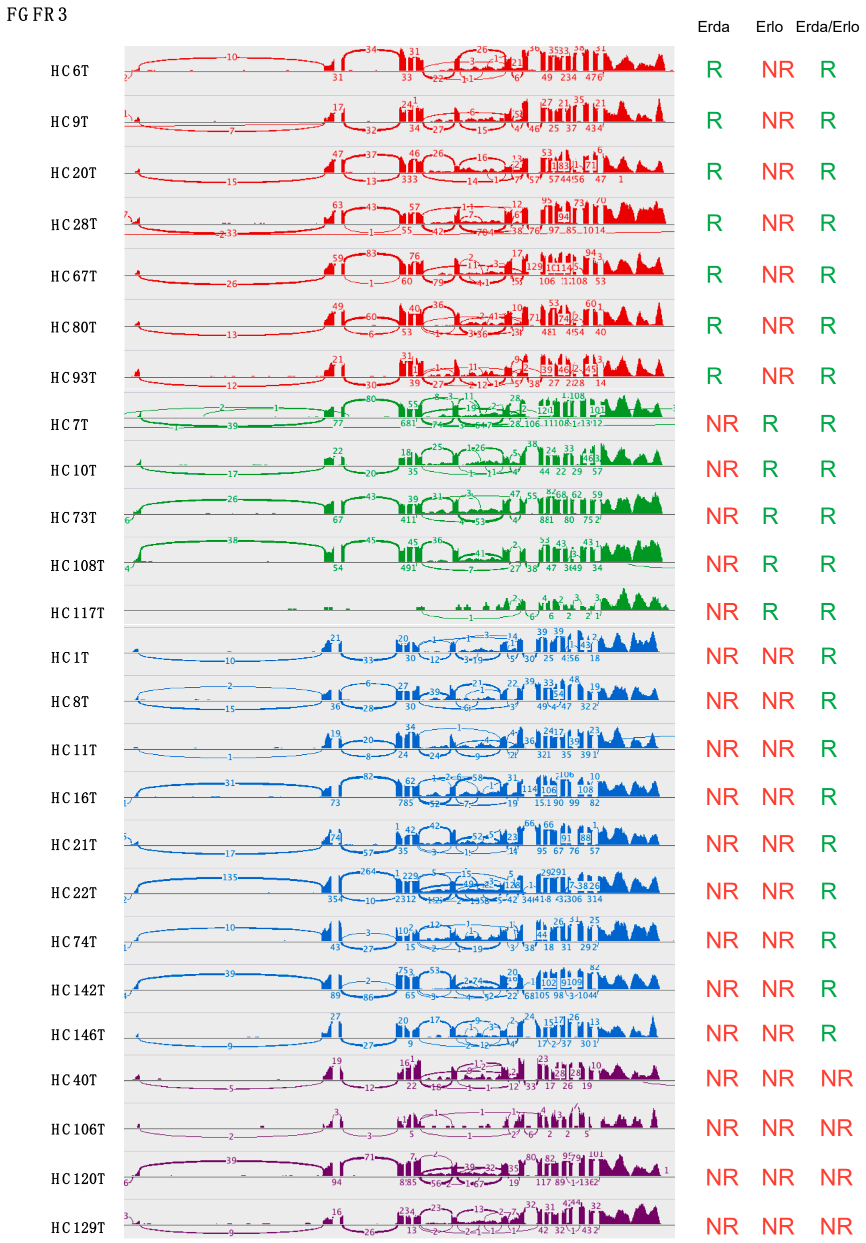

Figure S6. (**A**) Sashimi plots across RNA-seq samples of *FGFR4* mRNA in all 25 colorectal cancer TIC-spheroid lines. Read densities across exons are quantified in RPKM (read per kilobase/million) and junction reads are shown as arcs, annotated with read numbers. Abbreviations R and NR stand for "responsive" and "non-responsive", respectively, to erdafitinib, erlotinib, and the erdafitinib/erlotinib combination. (**B**) Sashimi plots across RNA-seq samples of *FGFR3* mRNA in all 25 colorectal cancer TIC-spheroid lines. Read densities across exons are quantified in RPKM (read per kilobase/million) and junction reads are shown as arcs, annotated with read numbers. Abbreviations R and NR stand for "responsive" and "non-responsive", respectively, to erdafitinib, erlotinib, and the erdafitinib/erlotinib combination.

Table S1. Mutational status detected by targeted next-generation sequencing of 409 cancer-related genes in each spheroid sample.

Chrom	Position	Ref	Variant	Frequency	Type	Allele Name	Gene ID	AAChange.refGene
HC1T								
chr2	5833842	C	T	54.4	SNP	---	*SOX11*	NM_003108:exon1:c.989C>T:p.A330V
chr3	178936091	G	A	47.8	SNP	COSM763	*PIK3CA*	NM_006218:exon10:c.1633G>A:p.E545K
chr5	112175513	G	T	46.3	SNP	COSM18822	*APC*	NM_000038:exon16:c.4222G>T:p.E1408X
chr7	50367288	C	T	44.4	SNP	---	*IKZF1*	NM_006060:exon3:c.95C>T:p.P32L
chr8	71037056	G	A	51.5	SNP	---	*NCOA2*	NM_006540:exon20:c.3961C>T:p.P1321S
chr18	48604788	A	G	100	SNP	---	*SMAD4*	NM_005359:exon12:c.1610A>G:p.D537G
chr22	36689877	G	T	43.4	SNP	---	*MYH9*	NM_002473:exon29:c.3870C>A:p.S1290R
HC6T								
chr5	112174920	AT	-	53.3	DEL	---	*APC*	NM_000038:exon16:c.3629_3630del:p.H1210fs
chr5	112175466	C	A	34.4	SNP	---	*APC*	NM_000038:exon16:c.4175C>A:p.S1392X
chr9	8340383	G	T	29.5	SNP	---	*PTPRD*	NM_130391:exon27:c.3992C>A:p.T1331N
chr17	7577121	G	A	99.9	SNP	COSM10659	*TP53*	NM_000546:exon8:c.817C>T:p.R273C
chrX	41031129	G	C	20.5	SNP	---	*USP9X*	NM_001039590:exon21:c.3066G>C:p.W1022C
HC7T								
chr2	5833373	G	A	50.4	SNP	---	*SOX11*	NM_003108:exon1:c.520G>A:p.A174T
chr4	88047313	C	T	100	SNP	---	*AFF1*	NM_005935:exon13:c.2615C>T:p.S872L
chr6	56483001	G	A	27.6	SNP	---	*DST*	NM_001723:exon23:c.5831C>T:p.A1944V
chr11	44129747	A	G	34.1	SNP	---	*EXT2*	NM_000401:exon2:c.584A>G:p.Q195R
chr11	118375555	G	A	69	SNP	---	*MLL*	NM_005933:exon27:c.8939G>A:p.G2980E
chr17	7577498	C	T	100	SNP	COSM43571	*TP53*	Splicing
chr17	12032474	C	T	100	SNP	---	*MAP2K4*	NM_003010:exon9:c.910C>T:p.R304X
chr17	29483091	T	-	33.5	DEL	---	*NF1*	NM_000267:exon2:c.151delT:p.F51fs
chr19	1615470	G	A	35.6	SNP	---	*TCF3*	NM_003200:exon18:c.1636C>T:p.R546W
chrX	63411291	G	A	52.9	SNP	---	*FAM123B*	NM_152424:exon2:c.1876C>T:p.R626X
HC9T								
chr3	10191645	A	G	67.3	SNP	---	*VHL*	NM_000551:exon3:c.638A>G:p.D213G
chr5	112164664	A	-	100	DEL	---	*APC*	NM_000038:exon14:c.1742delA:p.K581fs
chr17	7578247	-	A	100	INS	---	*TP53*	NM_000546:exon6:c.601dupT:p.L201fs
chr17	7579714	C	T	100	SNP	---	*TP53*	NM_000546:exon3:c.82G>A:p.E28K
HC10T								
chr3	37860464	T	C	49.3	SNP	---	*ITGA9*	NM_002207:exon28:c.3092T>C:p.V1031A
chr3	70014040	C	G	51	SNP	---	*MITF*	NM_000248:exon9:c.901C>G:p.P301A
chr3	89391088	G	A	48.5	SNP	---	*EPHA3*	NM_005233:exon5:c.1154G>A:p.R385Q
chr5	112162891	C	T	49.5	SNP	COSM29364	*APC*	NM_000038:exon12:c.1495C>T:p.R499X
chr5	112175639	C	T	49.2	SNP	COSM13127	*APC*	NM_000038:exon16:c.4348C>T:p.R1450X
chr11	106558429	A	C	51.1	SNP	---	*GUCY1A2*	NM_000855:exon8:c.2045T>G:p.L682R
chr12	49420286	C	A	38.8	SNP	---	*MLL2*	NM_003482:exon48:c.15463G>T:p.V5155F
chr16	68867358	T	C	48.6	SNP	---	*CDH1*	NM_004360:exon16:c.2605T>C:p.F869L
chr17	7577138	C	T	50.9	SNP	COSM43923	*TP53*	NM_000546:exon8:c.800G>A:p.R267Q
HC11T								
chr1	115053393	-	GCTGGA	45.5	INS	---	*TRIM33*	NM_015906:exon1:c.305_306insTCCAGC:p.A102delinsAPA
chr5	112175897	G	T	98.4	SNP	COSM19056	*APC*	NM_000038:exon16:c.4606G>T:p.E1536X
chr7	106509426	C	G	70.4	SNP	---	*PIK3CG*	NM_002649:exon2:c.1420C>G:p.L474V
chr10	96522463	A	G	49.9	SNP	---	*CYP2C19*	NM_000769:exon1:c.1A>G:p.M1V
chr11	95826309	C	A	52.9	SNP	---	*MAML2*	NM_032427:exon2:c.886G>T:p.D296Y
chr12	4398151	G	A	45.3	SNP	---	*CCND2*	NM_001759:exon4:c.715G>A:p.D239N
chr13	41240075	G	A	35.7	SNP	---	*FOXO1*	NM_002015:exon1:c.275C>T:p.A92V
chr17	5462049	G	A	100	SNP	---	*NLRP1*	NM_014922:exon4:c.1967C>T:p.T656M
chr17	7578398	G	-	100	DEL	COSM44659	*TP53*	NM_000546:exon5:c.532delC:p.H178fs
chr18	45368213	G	T	100	SNP	---	*SMAD2*	NM_005901:exon11:c.1389C>A:p.C463X
chrX	48650332	C	T	99.1	SNP	---	*GATA1*	NM_002049:exon3:c.302C>T:p.T101M
HC16T								
chr1	27088788	-	G	74.9	INS	---	*ARID1A*	NM_006015:exon7:c.2397dupG:p.Q799fs
chr4	153249384	C	T	20.6	SNP	COSM117308	*FBXW7*	NM_018315:exon10:c.1505C>T:p.S502L
chr4	153245446	G	A	79.2	SNP	COSM22979	*FBXW7*	NM_018315:exon8:c.1154G>A:p.R385H
chr5	112128191	C	T	100	SNP	COSM13130	*APC*	NM_000038:exon7:c.694C>T:p.R232X
chr8	145740393	G	A	79.5	SNP	---	*RECQL4*	NM_004260:exon9:c.1547C>T:p.A516V
chr12	49424728	C	T	35.5	SNP	---	*MLL2*	NM_003482:exon40:c.13619G>A:p.R4540Q
chr14	99640652	G	A	72.8	SNP	---	*BCL11B*	NM_022898:exon3:c.2308C>T:p.R770C
chr17	7577082	C	T	98.7	SNP	COSM99924	*TP53*	NM_000546:exon8:c.856G>A:p.E286K
HC20T								
chr6	51751973	G	A	50.3	SNP	---	*PKHD1*	NM_138694:exon44:c.7067C>T:p.P2356L
chr8	113569067	G	C	21.8	SNP	---	*CSMD3*	NM_052900:exon24:c.3847C>G:p.L1283V
chr8	113569114	T	C	31.2	SNP	---	*CSMD3*	NM_052900:exon24:c.3800A>G:p.H1267R
chr10	114920414	G	A	50.4	SNP	---	*TCF7L2*	NM_001146274:exon13:c.1355G>A:p.G452E
chr16	2124279	A	T	20.1	SNP	---	*TSC2*	NM_000548:exon22:c.2434A>T:p.S812C
chr17	7579389	G	A	100	SNP	---	*TP53*	NM_000546:exon4:c.298C>T:p.Q100X
chr18	48593504	G	T	100	SNP	---	*SMAD4*	NM_005359:exon10:c.1255G>T:p.G419W
HC21T								
chr4	153251894	T	C	52.6	SNP	---	*FBXW7*	NM_018315:exon6:c.872A>G:p.K291R
chr5	112175068	AC	-	72.3	DEL	---	*APC*	NM_000038:exon16:c.3777_3778del:p.I1259fs
chr7	138252340	C	A	24.8	SNP	---	*TRIM24*	NM_003852:exon10:c.1543C>A:p.Q515K
chr8	113317138	G	T	20	SNP	---	*CSMD3*	NM_198123:exon52:c.8078C>A:p.T2693K

Table S1. *Cont.*

chr9	135787745	G	C	55.5	SNP	---	*TSC1*	NM_000368:exon9:c.837C>G:p.H279Q
chr10	76735968	T	G	37.6	SNP	---	*KAT6B*	NM_012330:exon8:c.1873T>G:p.F625V
chr11	71715775	T	C	53.2	SNP	---	*NUMA1*	NM_006185:exon24:c.5917A>G:p.I1973V
chr17	7578275	G	A	99	SNP	COSM10733	*TP53*	NM_000546:exon6:c.574C>T:p.Q192X
HC22T								
chr4	106156877	A	T	96.4	SNP	---	*TET2*	NM_017628:exon3:c.1778A>T:p.Q593L
chr5	112162870	CACTACAGTATTA	-	100	DEL	---	*APC*	NM_000038:exon12:c.1474_1486del:p.H492fs
chr7	91603046	A	G	20.9	SNP	---	*AKAP9*	NM_005751:exon2:c.70A>G:p.K24E
chr17	7577548	C	T	100	SNP	COSM6932	*TP53*	NM_000546:exon7:c.733G>A:p.G245S
chr18	50848557	T	G	35.4	SNP	---	*DCC*	NM_005215:exon17:c.2525C>A:p.T842N
chr22	41553373	C	G	27.5	SNP	---	*EP300*	NM_001429:exon18:c.3462C>G:p.D1154E
HC28T								
chr3	71026184	C	A	51.4	SNP	---	*FOXP1*	NM_032682:exon17:c.1438G>T:p.E480X
chr3	178936082	G	A	52.2	SNP	COSM760	*PIK3CA*	NM_006218:exon10:c.1624G>A:p.E542K
chr5	112151261	C	T	100	SNP	COSM13862	*APC*	NM_000038:exon9:c.904C>T:p.R302X
chr22	24176338	C	T	54	SNP	---	*SMARCB1*	NM_003073:exon9:c.1129C>T:p.R377C
HC40T								
chr3	178936091	G	A	67.8	SNP	COSM125370	*PIK3CA*	NM_006218:exon10:c.1633G>A:p.E545K
chr5	112103086	AG	-	36.6	DEL	COSM25153	*APC*	NM_000038:exon4:c.417_418del:p.K139fs
chr5	112176398	G	A	60	SNP	---	*APC*	NM_000038:exon16:c.5107G>A:p.G1703R
chr5	176618907	C	T	65.6	SNP	---	*NSD1*	NM_022455:exon3:c.950C>T:p.T317M
chr7	55211097	G	A	42.5	SNP	---	*EGFR*	NM_005228:exon3:c.340G>A:p.E114K
chr10	70332760	G	T	48.4	SNP	---	*TET1*	NM_030625:exon2:c.665G>T:p.R222L
chr10	90767542	A	C	50.2	SNP	---	*FAS*	NM_000043:exon3:c.282A>C:p.K94N
chr13	28964142	A	T	31.7	SNP	---	*FLT1*	NM_002019:exon13:c.1760T>A:p.V587D
chr16	2127724	G	A	44.5	SNP	---	*TSC2*	NM_000548:exon26:c.2963G>A:p.R988H
chr16	27460579	C	G	28.3	SNP	---	*IL21R*	NM_021798:exon9:c.1592C>G:p.S531W
chr17	7578406	C	T	100	SNP	COSM99914	*TP53*	NM_000546:exon5:c.524G>A:p.R175H
chrX	41029432	A	C	45.8	SNP	---	*USP9X*	NM_001039590:exon19:c.2821A>C:p.I941L
HC67T								
chr5	38481794	C	T	76.2	SNP	---	*LIFR*	NM_002310:exon20:c.3197G>A:p.R1066Q
chr5	112175958	-	A	100	INS	COSM18561	*APC*	NM_000038:exon16:c.4662dupA:p.E1554fs
chr6	51524309	T	C	31	SNP	---	*PKHD1*	NM_138694:exon61:c.10615A>G:p.M3539V
chr6	152599456	C	T	65.6	SNP	---	*SYNE1*	NM_033071:exon45:c.6726A>C:p.E2242D
chr10	88649927	T	A	95.6	SNP	---	*BMPR1A*	NM_004329:exon4:c.176T>A:p.L59X
chr17	78360208	A	G	100	SNP	---	*RNF213*	NM_001256071:exon62:c.14698A>G:p.K4900E
HC73T								
chr5	112162891	C	T	57.8	SNP	COSM29364	*APC*	NM_000038:exon12:c.1495C>T:p.R499X
chr5	112175510	AG	-	47.2	DEL	COSM19088	*APC*	NM_000038:exon16:c.4217_4218del:p.Q1406fs
chr8	113564823	C	T	99.3	SNP	---	*CSMD3*	NM_052900:exon40:c.6069delT:p.F2023fs
chr13	110436398	C	G	68.7	SNP	---	*IRS2*	NM_003749:exon1:c.2003G>C:p.G668A
chr17	7578190	T	C	100	SNP	COSM99720	*TP53*	NM_000546:exon6:c.659A>G:p.Y220C
chr17	37881392	A	T	48.9	SNP	---	*ERBB2*	NM_004448:exon21:c.2584A>T:p.T862S
HC74T								
chr2	30143052	G	-	48.2	DEL	---	*ALK*	NM_004304:exon1:c.475G>A:p.G159R
chr5	112116592	C	T	51.3	SNP	COSM13134	*APC*	NM_000038:exon16:c.4185delT:p.S1395fs
chr5	112175476	T	-	50.2	DEL	---	*APC*	NM_000038:exon6:c.637C>T:p.R213X
chr7	92733555	A	C	30.3	SNP	---	*SAMD9*	NM_017654:exon3:c.1856T>G:p.L619R
chr13	113975779	G	A	48.3	SNP	---	*LAMP1*	NM_005561:exon7:c.937G>A:p.A313T
chr17	7577105	G	A	97.7	SNP	COSM10863	*TP53*	NM_000546:exon8:c.833C>T:p.P278L
chr17	48263150	C	T	53.6	SNP	---	*COL1A1*	NM_000088:exon50:c.4237G>A:p.D1413N
HC80T								
chr1	27106006	C	-	60	DEL	---	*ARID1A*	NM_006015:exon20:c.5617delC:p.P1873fs
chr5	112102946	G	A	36.9	SNP	---	*APC*	NM_000038:exon4:c.281G>A:p.R94H
chr5	112128143	C	T	99.2	SNP	COSM98420	*APC*	NM_000038:exon7:c.646C>T:p.R216X
chr7	142568063	C	T	27.9	SNP	---	*EPHB6*	NM_004445:exon15:c.2704C>T:p.R902C
chr12	43925980	G	A	47	SNP	---	*ADAMTS20*	NM_025003:exon3:c.472C>T:p.Q158X
chr12	56482415	G	C	27.6	SNP	---	*ERBB3*	NM_001982:exon8:c.963G>C:p.E321D
chr17	7577124	C	T	100	SNP	COSM10891	*TP53*	NM_000546:exon8:c.814G>A:p.V272M
HC93T								
chr1	27088705	T	-	25	DEL	---	*ARID1A*	NM_006015:exon7:c.2314delT:p.S772fs
chr3	52713613	AA	-	59.5	DEL	---	*PBRM1*	NM_018313:exon2:c.114_115del:p.L38fs
chr6	152642901	T	C	63.6	SNP	---	*SYNE1*	NM_033071:exon54:c.8395C>T:p.R2799C
chr17	7578406	C	T	100	SNP	COSM10648	*TP53*	NM_000546:exon5:c.524G>A:p.R175H
HC108T								
chr2	141356273	G	T	45.5	SNP	---	*LRP1B*	NM_018557:exon43:c.7121C>A:p.A2374E
chr3	142241655	T	C	69.6	SNP	---	*ATR*	NM_001184:exon23:c.4181A>G:p.Y1394C
chr5	112162891	C	T	67.7	SNP	COSM29364	*APC*	NM_000038:exon12:c.1495C>T:p.R499X
chr5	112175482	-	GAGTCGT	32.2	INS	---	*APC*	NM_000038:exon16:c.4190_4191insGAGTCGT:p.E1397fs
chr17	7578406	C	T	100	SNP	COSM10648	*TP53*	NM_000546:exon5:c.524G>A:p.R175H
HC117T								
chr5	112164586	C	T	40.4	SNP	COSM19040	*APC*	NM_000038:exon14:c.1660C>T:p.R554X
chr5	112175136	C	G	44.6	SNP	---	*APC*	NM_000038:exon16:c.3845C>G:p.S1282X
chr6	31133815	T	C	46.5	SNP	---	*POU5F1*	NM_002701:exon2:c.415A>G:p.I139V
chr8	42176918	G	A	23.1	SNP	---	*IKBKB*	NM_001556:exon14:c.1495G>A:p.E499K

Table S1. *Cont.*

chr11	44129720	T	C	53.2	SNP	---	*EXT2*	NM_000401:exon2:c.557T>C:p.F186S
chr17	7577121	G	A	97.6	SNP	COSM10659	*TP53*	NM_000546:exon8:c.817C>T:p.R273C
HC120T								
chr1	186301460	C	G	100	SNP	---	*TPR*	NM_003292:exon38:c.5471G>C:p.S1824T
chr2	29606678	C	T	25.1	SNP	---	*ALK*	NM_004304:exon5:c.1202G>A:p.R401Q
chr7	50467988	G	A	45.8	SNP	---	*IKZF1*	NM_006060:exon8:c.1223G>A:p.R408H
chr17	7578189	-	A	100	INS	---	*TP53*	NM_000546:exon6:c.660dupT:p.E221_P222delinsX
chr18	22804908	T	A	100	SNP	---	*ZNF521*	NM_015461:exon4:c.2974A>T:p.I992F
HC129T								
chr2	100627984	G	A	22.7	SNP	---	*AFF3*	NM_001025108:exon3:c.103C>T:p.Q35X
chr5	112175217	AAAAG	-	100	DEL	COSM18701	*APC*	NM_000038:exon16:c.3921_3925del:p.I1307fs
chr8	38271150	C	T	30.7	SNP	---	*FGFR1*	NM_015850:exon18:c.2459G>A:p.R820H
chr14	102551042	G	C	26.5	SNP	---	*HSP90AA1*	NM_005348:exon5:c.957C>G:p.D319E
chr15	40915657	A	-	24.3	DEL	---	*CASC5*	NM_144508:exon10:c.3195delA:p.R1065fs
chr17	7578406	C	T	100	SNP	COSM99022	*TP53*	NM_000546:exon5:c.524G>A:p.R175H
HC142T								
chr1	204403666	C	T	41.2	SNP	---	*PIK3C2B*	NM_002646:exon25:c.3587G>A:p.R1196Q
chr4	62801716	G	A	46.1	SNP	---	*LPHN3*	NM_015236:exon14:c.2168G>A:p.S723N
chr6	44219235	AA	-	50	DEL	---	*HSP90AB1*	NM_007355:exon8:c.1204_1205del:p.K402fs
chr6	152485403	G	T	43.7	SNP	---	*SYNE1*	NM_033071:exon130:c.23472C>A:p.S7824R
chr9	37002728	G	A	32.3	SNP	---	*PAX5*	NM_016734:exon5:c.521C>T:p.S174L
chr11	108188143	T	G	86.7	SNP	---	*ATM*	NM_000051:exon43:c.6242T>G:p.L2081X
HC146T								
chr1	27105520	G	-	47.5	DEL	---	*ARID1A*	NM_006015:exon20:c.5131delG:p.G1711fs
chr1	186645619	G	A	46.4	SNP	---	*PTGS2*	NM_000963:exon7:c.950C>T:p.T317I
chr7	13971180	A	G	48.7	SNP	---	*ETV1*	NM_004956:exon9:c.749T>C:p.F250S
chr17	7578555	C	T	100	SNP	---	*TP53*	Splicing

Table S2. List of 409 cancer-related genes investigated in the present study.

ABL1	*CBL*	*EP300*	*GATA2*	*LAMP1*	*MYD88*	*PKHD1*	*SMARCA4*	*WHSC1*
ABL2	*CCND1*	*EP400*	*GATA3*	*LCK*	*MYH11*	*PLAG1*	*SMARCB1*	*WRN*
ACVR2A	*CCND2*	*EPHA3*	*GDNF*	*LIFR*	*MYH9*	*PLCG1*	*SMO*	*WT1*
ADAMTS20	*CCNE1*	*EPHA7*	*GNA11*	*LPHN3*	*NBN*	*PLEKHG5*	*SMUG1*	*XPA*
AFF1	*CD79A*	*EPHB1*	*GNAQ*	*POT1*	*NCOA1*	*PML*	*SOCS1*	*XPC*
AFF3	*CD79B*	*EPHB4*	*GNAS*	*LPP*	*NCOA2*	*PMS1*	*SOX11*	*XPO1*
AKAP9	*CDC73*	*EPHB6*	*GPR124*	*LRP1B*	*NCOA4*	*PMS2*	*SOX2*	*XRCC2*
AKT1	*CDH1*	*ERBB2*	*GRM8*	*LTF*	*NF1*	*POU5F1*	*SRC*	*ZNF384*
AKT2	*CDH11*	*ERBB3*	*GUCY1A2*	*LTK*	*NF2*	*PPARG*	*SSX1*	*ZNF521*
AKT3	*CDH2*	*ERBB4*	*HCAR1*	*MAF*	*NFE2L2*	*PPP2R1A*	*STK11*	
ALK	*CDH20*	*ERCC1*	*HIF1A*	*MAFB*	*NFKB1*	*PRDM1*	*STK36*	
APC	*CDH5*	*ERCC2*	*HLF*	*MAGEA1*	*NFKB2*	*PRKAR1A*	*SUFU*	
AR	*CDK12*	*ERCC3*	*HNF1A*	*MAGI1*	*NIN*	*PRKDC*	*SYK*	
ARID1A	*CDK4*	*ERCC4*	*HOOK3*	*MALT1*	*NKX2-1*	*PSIP1*	*SYNE1*	
ARID2	*CDK6*	*ERCC5*	*HRAS*	*MAML2*	*NLRP1*	*PTCH1*	*TAF1*	
ARNT	*CDK8*	*ERG*	*HSP90AA1*	*MAP2K1*	*NOTCH1*	*PTEN*	*TAF1L*	
ASXL1	*CDKN2A*	*ESR1*	*HSP90AB1*	*MAP2K2*	*NOTCH2*	*PTGS2*	*TAL1*	
ATF1	*CDKN2B*	*ETS1*	*ICK*	*MAP2K4*	*NOTCH4*	*PTPN11*	*TBX22*	
ATM	*CDKN2C*	*ETV1*	*IDH1*	*MAP3K7*	*NPM1*	*PTPRD*	*TCF12*	
ATR	*CEBPA*	*ETV4*	*IDH2*	*MAPK1*	*NRAS*	*PTPRT*	*TCF3*	
ATRX	*CHEK1*	*EXT1*	*IGF1R*	*MAPK8*	*NSD1*	*RAD50*	*TCF7L1*	
AURKA	*CHEK2*	*EXT2*	*IGF2*	*MARK1*	*NTRK1*	*RAF1*	*TCF7L2*	
AURKB	*CIC*	*EZH2*	*IGF2R*	*MARK4*	*NTRK3*	*RALGDS*	*TCL1A*	
AURKC	*CKS1B*	*FAM123B*	*IKBKB*	*MBD1*	*NUMA1*	*RARA*	*TET1*	
AXL	*CMPK1*	*FANCA*	*IKBKE*	*MCL1*	*NUP214*	*RB1*	*TET2*	
BAI3	*COL1A1*	*FANCC*	*IKZF1*	*MDM2*	*NUP98*	*RECQL4*	*TFE3*	
BAP1	*CRBN*	*FANCD2*	*IL2*	*MDM4*	*PAK3*	*REL*	*TGFBR2*	
BCL10	*CREB1*	*FANCF*	*IL21R*	*MEN1*	*PALB2*	*RET*	*TGM7*	
BCL11A	*CREBBP*	*FANCG*	*IL6ST*	*MET*	*PARP1*	*RHOH*	*THBS1*	
BCL11B	*CRKL*	*FAS*	*IL7R*	*MITF*	*PAX3*	*RNASEL*	*TIMP3*	
BCL2	*CRTC1*	*FBXW7*	*ING4*	*MLH1*	*PAX5*	*RNF2*	*TLR4*	
BCL2L1	*CSF1R*	*FGFR1*	*IRF4*	*MLL*	*PAX7*	*RNF213*	*TLX1*	
BCL2L2	*CSMD3*	*FGFR2*	*IRS2*	*MLL2*	*PAX8*	*ROS1*	*TNFAIP3*	
BCL3	*CTNNA1*	*FGFR3*	*ITGA10*	*MLL3*	*PBRM1*	*RPS6KA2*	*TNFRSF14*	
BCL6	*CTNNB1*	*FGFR4*	*ITGA9*	*MLLT10*	*PBX1*	*RRM1*	*TNK2*	
BCL9	*CYLD*	*FH*	*ITGB2*	*MMP2*	*PDE4DIP*	*RUNX1*	*TOP1*	
BCR	*CYP2C19*	*FLCN*	*ITGB3*	*MN1*	*PDGFB*	*RUNX1T1*	*TP53*	
BIRC2	*CYP2D6*	*FLI1*	*JAK1*	*MPL*	*PDGFRA*	*SAMD9*	*TPR*	
BIRC3	*DAXX*	*FLT1*	*JAK2*	*MRE11A*	*PDGFRB*	*SBDS*	*TRIM24*	
BIRC5	*DCC*	*FLT3*	*JAK3*	*MSH2*	*PER1*	*SDHA*	*TRIM33*	
BLM	*DDB2*	*FLT4*	*JUN*	*MSH6*	*PGAP3*	*SDHB*	*TRIP11*	
BLNK	*DDIT3*	*FN1*	*KAT6A*	*MTOR*	*PHOX2B*	*SDHC*	*TRRAP*	
BMPR1A	*DDR2*	*FOXL2*	*KAT6B*	*MTR*	*PIK3C2B*	*SDHD*	*TSC1*	
BRAF	*DEK*	*FOXO1*	*KDM5C*	*MTRR*	*PIK3CA*	*SEPT9*	*TSC2*	
BRD3	*DICER1*	*FOXO3*	*KDM6A*	*MUC1*	*PIK3CB*	*SETD2*	*TSHR*	
BRIP1	*DNMT3A*	*FOXP1*	*KDR*	*MUTYH*	*PIK3CD*	*SF3B1*	*UBR5*	
BTK	*DPYD*	*FOXP4*	*KEAP1*	*MYB*	*PIK3CG*	*SGK1*	*UGT1A1*	
BUB1B	*DST*	*FZR1*	*KIT*	*MYC*	*PIK3R1*	*SH2D1A*	*USP9X*	
CARD11	*EGFR*	*G6PD*	*KLF6*	*MYCL1*	*PIK3R2*	*SMAD2*	*VHL*	
CASC5	*EML4*	*GATA1*	*KRAS*	*MYCN*	*PIM1*	*SMAD4*	*WAS*	

© 2020 by the authors. Licensee MDPI, Basel, Switzerland. This article is an open access article distributed under the terms and conditions of the Creative Commons Attribution (CC BY) license (http://creativecommons.org/licenses/by/4.0/).
